# Searching for a Paradigm Shift in Auger-Electron Cancer Therapy with Tumor-Specific Radiopeptides Targeting the Mitochondria and/or the Cell Nucleus

**DOI:** 10.3390/ijms23137238

**Published:** 2022-06-29

**Authors:** Célia Fernandes, Elisa Palma, Francisco Silva, Ana Belchior, Catarina I. G. Pinto, Joana F. Guerreiro, Hugo M. Botelho, Filipa Mendes, Paula Raposinho, António Paulo

**Affiliations:** 1 Centro de Ciências e Tecnologias Nucleares, Instituto Superior Técnico, Universidade de Lisboa, Campus Tecnológico e Nuclear, Estrada Nacional 10, Km 139.7, 2695-066 Bobadela LRS, Portugal; celiaf@ctn.tecnico.ulisboa.pt (C.F.); elisa@ctn.tecnico.ulisboa.pt (E.P.); fsilva@ctn.tecnico.ulisboa.pt (F.S.); anabelchior@tecnico.ulisboa.pt (A.B.); catarina.pinto@tecnico.ulisboa.pt (C.I.G.P.); joanaguerreiro@ctn.tecnico.ulisboa.pt (J.F.G.); fmendes@ctn.tecnico.ulisboa.pt (F.M.); 2 Departamento de Engenharia e Ciências Nucleares, Instituto Superior Técnico, Universidade de Lisboa, 1749-016 Lisboa, Portugal; 3 BioISI—Instituto de Biosistemas e Ciências Integrativas, Faculdade de Ciências, Universidade de Lisboa, 1749-016 Lisboa, Portugal; hmbotelho@fc.ul.pt

**Keywords:** radiopharmaceuticals, Targeted Radionuclide Therapy (TRT), auger electron emitters, technetium-99m, DNA intercalators, mitochondrion-tropic probes

## Abstract

Although ^99m^Tc is not an ideal Auger electron (AE) emitter for Targeted Radionuclide Therapy (TRT) due to its relatively low Auger electron yield, it can be considered a readily available “model” radionuclide useful to validate the design of new classes of AE-emitting radioconjugates. With this in mind, we performed a detailed study of the radiobiological effects and mechanisms of cell death induced by the dual-targeted radioconjugates **^99m^Tc-TPP-BBN** and **^99m^Tc-AO-BBN** (TPP = triphenylphosphonium; AO = acridine orange; BBN = bombesin derivative) in human prostate cancer PC3 cells. **^99m^Tc-TPP-BBN** and **^99m^Tc-AO-BBN** caused a remarkably high reduction of the survival of PC3 cells when compared with the single-targeted congener **^99m^Tc-BBN**, leading to an augmented formation of γH2AX foci and micronuclei. **^99m^Tc-TPP-BBN** also caused a reduction of the mtDNA copy number, although it enhanced the ATP production by PC3 cells. These differences can be attributed to the augmented uptake of **^99m^Tc-TPP-BBN** in the mitochondria and enhanced uptake of **^99m^Tc-AO-BBN** in the nucleus, allowing the irradiation of these radiosensitive organelles with the short path-length AEs emitted by ^99m^Tc. In particular, the results obtained for **^99m^Tc-TPP-BBN** reinforce the relevance of targeting the mitochondria to promote stronger radiobiological effects by AE-emitting radioconjugates.

## 1. Introduction

In the past few years, Targeted Radionuclide Therapy (TRT) with β^−^ emitters has shown significant progress, which has led to the recent approval of the somatostatin analog ^177^Lu-DOTATATE (LUTATHERA^®^) for clinical use in the treatment of neuroendocrine neoplasms. Additionally, the prostate-specific membrane antigen (PSMA) inhibitor ^177^Lu-PSMA-617 (PLUVICTO^TM^) has been recently approved by the FDA to treat metastatic castration-resistant prostate cancer (PCa) [1,2]. However, the use of β^−^ emitters in the TRT of cancer has several limitations, such as the nephrotoxicity and beta radiation resistance encountered in a non-negligible number of patients. Targeted alpha therapy (TAT) can be an alternative, and promising preclinical and clinical data were reported in a few instances [3]. Unfortunately, most alpha emitters (e.g., ^211^At, ^225^Ac and ^231^Bi) have a low availability that limits their clinical use. Auger electron (AE) emitters can be a more feasible alternative, because this class of radionuclides has an easier availability than alpha emitters, as many of them are already commonly used in nuclear medicine imaging (e.g., ^123^I, ^125^I, ^67^Ga, ^99m^Tc or ^111^In). Most relevantly, there is an increasing availability of new and more suitable AE emitters obtained through innovative production methods (e.g., ^161^Tb, ^195m^Pt, ^197^Hg, ^103m^Rh and ^165^Er) [4].

TRT using AE emitters presents the additional advantage of having potential in the treatment of small size cancers and metastases because of their high level of cytotoxicity, high linear energy transfer (LET) and short-range biological effectiveness. From a dosimetric point of view, the highest relative biological effectiveness (RBE) of the AE emitters results when these radionuclides are internalized in highly radiosensitive organelles, such as the cell nucleus and the mitochondria, or even when linked to the cell membrane [5,6,7]. A great challenge is to avoid the undue irradiation of nontarget tissues by γ-photons often emitted by AE-emitting radionuclides. The specific delivery of AE emitters to these radiosensitive organelles (nucleus or mitochondria) in cancer cells might enhance the radiotherapeutic effects at lower doses, thus minimizing any undesired side effects (e.g., hematological toxicity and kidney damage). Towards this goal, a variety of AE-emitting radionuclides have been studied in the past few years by several research groups, using a variety of chemical forms spanning from simple radiochemical precursors (e.g., ^99m^TcO_4_^−^ or ^201^TlCl) to target-specific compounds obtained based on small molecules, monoclonal antibodies or peptides recognizing antigens, such as, receptors or enzymes overexpressed in cancer cells [4,8,9].

Historically, ^125^I has played a pioneer role in the study of the therapeutic potential of AE emitters, mainly due to its easy incorporation into nucleotides (e.g., 5-[(^125^)I]iodo-2¢- deoxyuridine (^125^I-UdR)), which allowed to show that there is an inverse relationship between the ^125^I-distance to the DNA and DNA damage and cell killing [10]. Hence, the cell’s nucleus has been considered the most important subcellular target to obtain efficient AE therapy outcomes. Interestingly, it has been reported that the radioiodinated PSMA inhibitor [^125^I]I-DCIBzL has a high potential for clinical translation to treat prostate cancer, which was attributed to the perinuclear localization of this radioconjugate in PSMA-positive PCa cells [11,12,13].

The influence of the proximity to DNA and accumulation in the cell nucleus on DNA damage and other radiobiological effects was thoroughly investigated for several other AE emitters, namely SPECT radiometals in clinical use such as ^67^Ga, ^111^In or ^99m^Tc or less common but more efficient AE emitters such as ^195m^Pt and ^191^Pt [14,15]. For instance, Terry’s group demonstrated how the DNA damage induced by ^67^Ga and ^111^In depends on their proximity to the DNA using a cell-free plasmid DNA assay [16,17]. Several classical DNA intercalators or groove binders have been studied as nuclear-targeting vectors for AE-emitting radionuclides, such as radiolabeled derivatives of acridine orange (AO) [18], pyrene [19], DAPI [20] and doxorubicin [21]. Our group radiolabeled several AO derivatives with the AE emitters ^99m^Tc and ^125^I and demonstrated that some of the compounds homed to the nucleus, resulting in an increased number of double-stranded breaks (DSBs) [18]. In a similar way, Alberto’s group explored the effect of ^99m^Tc-labeled pyrene conjugates on cell survival and found that these compounds exhibited a DNA-damaging effect, leading to mitotic catastrophe [22]. The need for close association with DNA molecules for AE-generated DNA lesion formation was also demonstrated by Reissig [19] using related ^99m^Tc-labeled pyrene constructs carrying alkyne groups of variable lengths. 

Other strategies to carry AE emitters to the cell nucleus rely on the use of nuclear localization sequences (NLS) and cell-penetrating peptides. Among those studies, the anti-HER2 antibody trastuzumab was functionalized with two cationic peptides (NLS_5-10_ or TAT_1-3_) to improve the nuclear uptake in HER2-positive breast cancer, but this effect was limited and nonspecific [23]. Another approach for the in vivo targeting of DNA with AE emitters takes advantage of the nuclear trafficking properties of some cell surface receptors. Of particular interest are members of the human epidermal growth factor receptor (EGFR) family that contain NLS sequences in the transmembrane region. The Reilly group extensively explored this concept for nuclear targeting with AE emitters [4]. One of the studied radioconjugates ([^111^In]-In-DTPA-EGF) has come the closest to clinical translation [24]. However, its evaluation in patients did not confirm the promising results obtained in the animal models [25]. 

Although the nucleus and DNA have been considered the primary cellular targets of radiation damage, it has been shown that internalization into cancer cells and delivery to the cell nucleus is not required for cell killing with AE-emitting radionuclides. An important breakthrough was reported by Pouget and collaborators, who showed that targeting the cell membrane can be an effective strategy for killing cancer cells with AEs [7]. 

The mitochondrion is another interesting extranuclear target for ionizing radiation (IR)-based cancer therapies but remains relatively understudied when compared with the cell nucleus. However, circular mitochondrial DNA, like genomic DNA, is sensitive to IR-induced damage, being less prone to undergoing repair processes. Moreover, IR can alter the mitochondrial function, induce mitochondrial oxidative stress and cause mitochondrial-induced apoptosis, either by direct or indirect effects. Most studies have focused on mitochondria-targeted radiosensitizers for external beam radiation therapy (EBRT), using triphenylphosphonium (TPP) derivatives or small peptides [26]. In contrast, studies with mitotropic therapeutic radioconjugates, namely those carrying AE emitters, are scarce and almost limited to the work that we recently reported for the dual-targeted radioconjugate **^99m^Tc-TPP-BBN** (TPP = triphenylphosphonium; BBN = bombesin derivative) [27]. 

**Figure 1:**
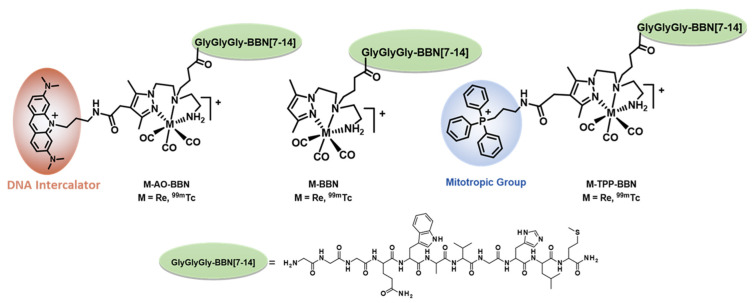
^99m^Tc-labeled BBN derivatives carrying or not organelle-specific pharmacophores evaluated in this work.

Although ^99m^Tc is not an ideal AE for TRT due to its relatively low Auger electron yield, it can be considered a readily available “model” radionuclide useful in validating the design of new classes of AE-emitting radioconjugates. Thus, with this in mind, we designed the dual-targeted **^99m^Tc-TPP-BBN** as an AE-emitting mitotropic therapeutic radioconjugate carrying: (i) a BBN sequence to target the gastrin-releasing peptide receptor (GRPR) and promote a selective uptake in GRPR (+) human prostate cancer PC3 cells and (ii) a TPP pharmacophore to provide an increased accumulation in the mitochondria of PC3 cells (Figure 1). Our initial studies showed that **^99m^Tc-TPP-BBN** caused a remarkably high reduction in the survival of human prostate cancer PC3 cells when compared with the single-targeted congener **^99m^Tc-BBN** [27]. This difference was attributed to the enhanced uptake of **^99m^Tc-TPP-BBN** in the mitochondria and the consequent irradiation of this radiosensitive organelle with the AEs emitted by ^99m^Tc, which present a high-energy deposition and ultra-short trajectories. Previously, other researchers have also argued that the differences in the radiobiological effects induced in a rat thyroid cell line by ^99m^TcO_4_^−^ and by the radiopharmaceuticals [^99m^Tc]Tc-hexamethyl-propylene-aminoxime (^99m^Tc-HMPAO) and [^99m^Tc]Tc-hexakis-2-methoxyisobutylisonitrile (^99m^Tc-MIBI) were due to their different subcellular distribution, namely in terms of mitochondrial accumulation [28].

These encouraging results obtained for **^99m^Tc-TPP-BBN** prompted us to further validate the relevance of mitochondrial targeting in AE cancer therapy. Towards this goal, we performed a more detailed study of the radiobiological effects induced by **^99m^Tc-TPP-BBN** in PC3 cells in comparison with a related dual-targeted **^99m^Tc-AO-BBN** (AO = acridine orange) (Figure 1). Instead of the TPP moiety, the latter contains a DNA intercalator to promote a preferential accumulation in the cell nucleus [5], the canonical target of radiotoxic effects by AE-emitting radionuclides. In this manuscript, we describe the subcellular localization of complexes **^99m^Tc-TPP-BBN** and **^99m^Tc-AO-BBN** to assess their targeting ability towards the nucleus or the mitochondria and report on the detailed study of the radiobiological effects exerted by each dual-targeted radioconjugate in PC3 cells. This study included the evaluation of the influence of **^99m^Tc-TPP-BBN** and **^99m^Tc-AO-BBN** in the cellular proliferation rate, nuclear DNA damage and mitochondrial function in comparison with **^99m^Tc-BBN**, which does not contain any organelle-specific pharmacophore.

## 2. Results and Discussion

### 2.1. Chemistry and Radiochemistry

Previously, we showed that the length of the methylenic linker (*n* = 3, 5 or 8) between the AO group and the fourth position of the pyrazolyl ring in the ^99m^Tc(I)-tricarbonyl complexes, anchored by tridentate pyrazolyldiamine chelators, has a strong influence on the DNA damage exerted by these AO-containing ^99m^Tc complexes [18,29]. This trend was found either in vitro in plasmid DNA or in vivo in PC3 cells and was attributed to the placement of ^99m^Tc at the shortest distance to the DNA for the complex with the shortest methylenic linker (propylenic; *n* = 3), upon AO intercalation into the double helix. Such a short distance enhanced the formation of DSBs by the short path-length AEs emitted by ^99m^Tc. 

Thus, we considered the same propylenic linker to attach the AO to the dual-targeted chelator **AO**-**Pz-BBN** used to obtain the **M-AO-BBN** (M = Re and ^99m^Tc) complexes evaluated in this work (Figure 1). As for the BBN peptide, it contains the G3-BBN [7-14] sequence with a triglycine (G3) linker cleavable by cathepsin B, as we recently demonstrated for the **Re-BBN** complex [27]. The presence of the G3 linker is crucial to obtain an augmented accumulation of ^99m^Tc or AO chromophore in the nucleus of PC3 cells for dual-targeted M(I) (M = Re and ^99m^Tc) tricarbonyl complexes similar to **M-AO-BBN**. Most probably, the G3 linker is cleaved by cathepsin B inside the cells, leading to a small-sized AO-containing Re(I)/^99m^Tc(I) fragment with a better ability to reach the cell nucleus.

**Scheme 1:**
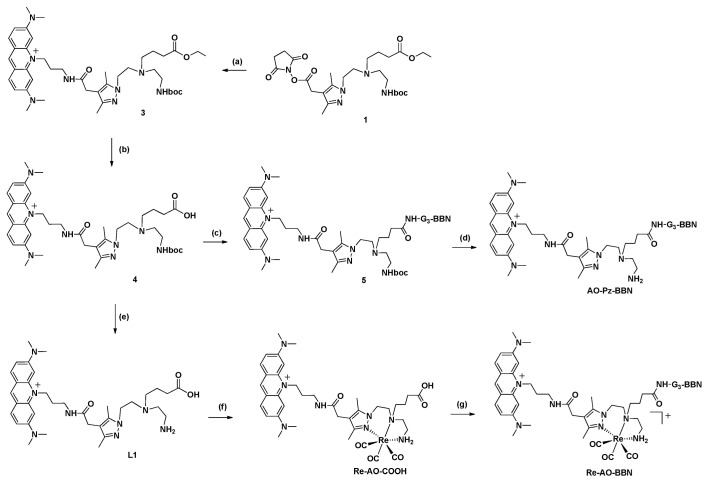
Synthesis of the BBN derivatives **AO**-**Pz-BBN** and **Re-AO-BBN**. (**a**) 3,6-bis(dimethylamino)-10-(3-(1,3-dioxoisoindolin-2-yl)-propyl)acridinium iodide (**2**), DIPEA, dry DMF, 3 days; (**b**) NaOH (aq.), 75 °C, overnight; (**c**) G3-BBN [7-14], DMF, DIPEA, HBTU, 4 h; (**d**) CH_2_Cl_2_, TFA, 1,2-Ethanedithiol (2.5 %), r.t., 2 h; (**e**) CH_2_Cl_2_, TFA, R.T., 5 h; (**f**) H_2_O, [Re(CO)_3_(H_2_O)_3_]Br, reflux, 12 h; (**g**) G3-BBN [7-14], DMF, DIPEA, HBTU, 3 h.

**Figure 2:**
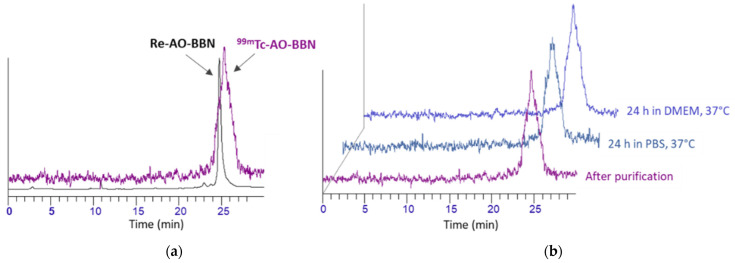
RP-HPLC radiochromatograms (γ-detection) of **^99m^Tc-AO-BBN**: (**a**) immediately after purification and in comparison with the congener **Re-AO-BBN** (UV detection); (**b**) after incubation in PBS pH 7.4 and DMEM cell culture at 37 °C for 24 h.

The dual-targeted bifunctional chelator **AO**-**Pz-BBN** was synthesized, as we have previously reported for similar compounds [30]. As shown in Scheme 1, the synthesis of **AO**-**Pz-BBN** was done in solution by the in situ activation of the pendant carboxylic acid function of a pyrazolyl diamine prochelator (compound **4**), followed by an amidation reaction with the terminal amino group from the G3-BBN [7-14] peptide. The synthesis of the respective pyrazolyldiamine Re(I) complex, **Re-AO**-**BBN**, was also performed in a solution by reacting the **Re-AO-COOH** complex with the G3-BBN [7-14] sequence (Scheme 1). The compounds **AO**-**Pz-BBN** and **Re-AO**-**BBN** were recovered by precipitation from the respective reaction mixtures with diethyl ether, followed by semipreparative HPLC purification of the crude solids. Their characterization was done by ESI-MS and analytical HPLC (Figure 2, Figures S1 and S2). The obtained ESI(+) mass spectra confirmed the formation of the desired conjugates being observed the expected molecular ion peaks with isotope distributions in agreement with the proposed formulations.

**Scheme 2:**
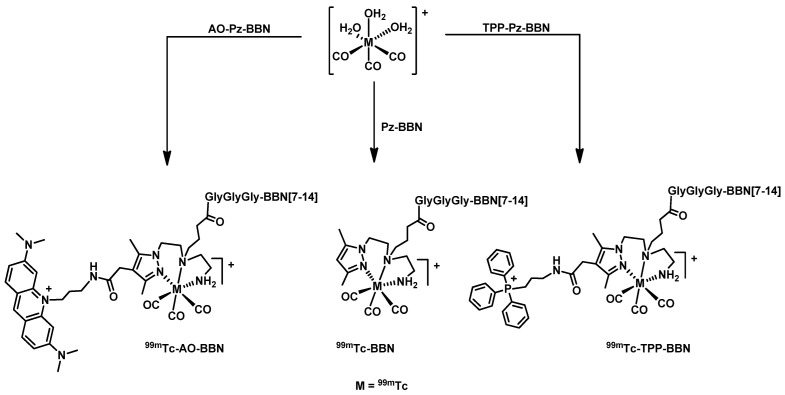
Synthesis of ^99m^Tc complexes: **^99m^Tc-AO-BBN**, **^99m^Tc-BBN** and **^99m^Tc-TPP-BBN**. Synthesis in aqueous solution pH = 7, 30 min, 100 °C; [AO-Pz-BBN]= 10^−4^ M; [Pz-BBN]= 5 × 10^−5^ M; [TPP-Pz-BBN] = 10^−4^ M.

The complex **^99m^Tc-AO-BBN** was synthesized by the reaction of the *fac*-[^99m^Tc(CO)_3_(H_2_O)_3_]^+^ precursor with the dual-targeted chelator **AO**-**Pz-BBN** in an aqueous medium (Scheme 2)**,** as we described previously for **^99m^Tc-BBN** and **^99m^Tc-TPP-BBN [27]**. All these ^99m^Tc complexes were purified by HPLC prior to their evaluation in the several biological studies reported in this work to separate the respective cold chelators. The chemical identification of the new complex **^99m^Tc-AO-BBN** was done by HPLC comparison with the Re congener (Figure 2). The characterization of **^99m^Tc-AO-BBN** also involved the assessment of its lipophilicity/hydrophilicity by determination of the n-octanol/PBS partition coefficient (P_o/w_) using the shake flask method (Table 1) [31]. The radiopeptide **^99m^Tc-AO-BBN** has a hydrophilic nature, showing a similar log P_o/w_ value (−0.84) with that exhibited by **^99m^Tc-BBN** (−0.75), in contrast with **^99m^Tc-TPP-BBN**, which is moderately lipophilic (log P_o/w_ = 0.45). This trend can be accounted for by a higher hydrophilic character for the AO moiety when compared with TPP, due to the presence of several tertiary amine groups in the aromatic scaffold of the former. 

The in vitro stability of **^99m^Tc-AO-BBN** was evaluated in the presence of PBS pH 7.4 and cell culture medium (DMEM) upon incubation of the complex at 37 °C for 24 h, corresponding to the maximum incubation time used in the biological assays. Aliquots of the mixtures were analyzed by RP-HPLC, and representative examples of the respective radiochromatograms are shown in Figure 2. As we previously reported for **^99m^Tc-BBN** [27], the complex **^99m^Tc-AO-BBN** remained stable in the presence of PBS and the cell culture medium.

### 2.2. Cellular Uptake and Binding Studies

**Figure 3:**
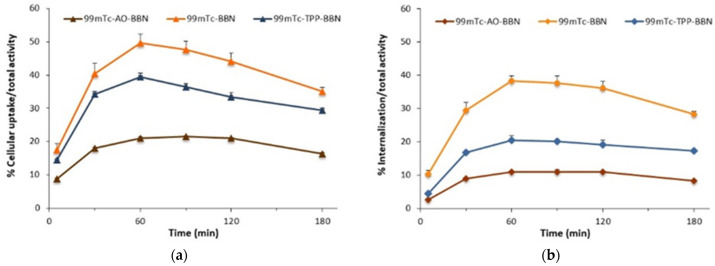

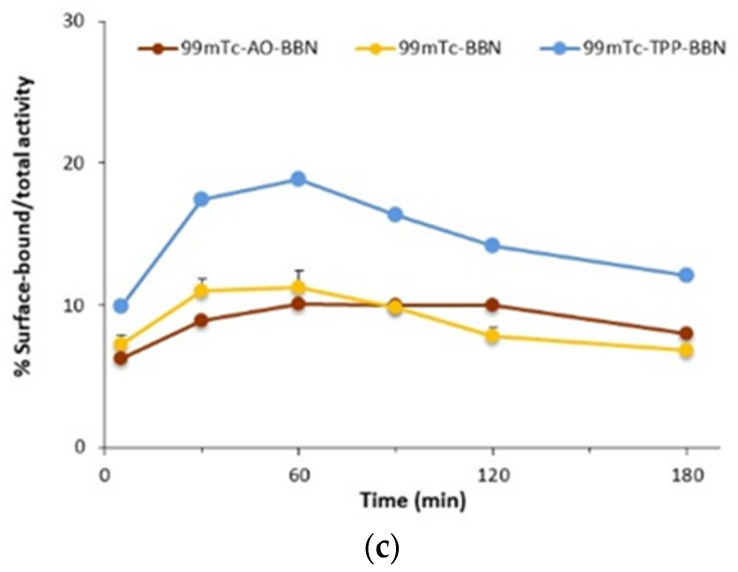
Time-dependent cellular uptake (**a**), internalization (**b**) and surface-bound (**c**) **^99m^Tc-BBN**, ^**99m**^**Tc-AO-BBN** and **^99m^Tc-TPP-BBN** in PC3 cells at 37 °C. The results are expressed as a percentage of the total (applied) activity and calculated from independent biological replicates (mean ± SEM, *n* = 4).

The cellular uptake of **^99m^Tc-AO-BBN** was studied in PC3 cells that overexpress GRPR, aiming to evaluate the ability of this dual-targeted complex to bind and internalize into the cells and to compare with the results previously reported by us for **^99m^Tc-BBN** and **^99m^Tc-TPP-BBN [27]**. The study was performed by incubating the PC3 cells with **^99m^Tc-AO-BBN** for different time intervals (5–180 min). For each time point, the overall radioactivity associated with the cells (cellular uptake) and that associated with the membrane, as well as that internalized in the cells, were determined by gamma-counting measurements. The results are presented in Figure 3 in comparison with those reported previously for **^99m^Tc-BBN** and **^99m^Tc-TPP-BBN [27]**.

As shown in Figure 3, the cellular uptake (Figure 3a) and internalization (Figure 3b) curves obtained for **^99m^Tc-AO-BBN** have a similar profile to those that we previously reported for **^99m^Tc-BBN** and **^99m^Tc-TPP-BBN** [27], where the maximum values (per applied radioactivity) were observed for the 1-h incubation time point. The maximum uptake and internalized values spanned the ranges 21.1–49.7% and 11.0–38.4%, respectively, a trend observed in both cases: **^99m^Tc-BBN** > **^99m^Tc-TPP-BBN > ^99m^Tc-AO-BBN**. Furthermore, a different trend was observed for the cell membrane binding (per applied activity) with **^99m^Tc-TPP-BBN**, showing the highest values (Figure 3c). However, considering the percentage of the total cell-associated activity that attached to the membrane, the dual-targeted complexes presented a higher tendency to bind to the cell membrane when compared with the single-targeted congener. For instance, at the 60-min time point, for which a maximum uptake value was observed for all radiocomplexes, roughly 50% of the total cell-associated **^99m^Tc-AO-BBN** and **^99m^Tc-TPP-BBN** were bound to the cell membrane against only ca. 20% for **^99m^Tc-BBN**. This behavior is certainly related to the larger overall positive charge of the dual-targeted complexes and/or presence of the intrinsically positive pharmacophores, favoring electrostatic interactions with the negatively charged cell membrane. 

Thereafter, we performed blockade assays with the GRPR agonist [Tyr^4^]-BBN to evaluate if the internalization of **^99m^Tc-AO-BBN** is mediated by the GRPR. For that, **^99m^Tc-AO-BBN** was incubated with PC3 cells for different time points (30 and 60 min) in the presence of a fixed concentration of cold [Tyr^4^]-BBN, as we previously described for **^99m^Tc-BBN** and **^99m^Tc-TPP-BBN**. The percentages of inhibition found for **^99m^Tc-AO-BBN** were 78% for both time points, corresponding to values that were intermediate between those determined for **^99m^Tc-BBN** (70–72%) and **^99m^Tc-TPP-BBN** (88–89%) under the same conditions (Figure S3) [27]. These data indicate that the interaction with the GRPR seems to be the dominant process in the internalization of the different radiocomplexes, including for the dual-targeted ones. 

The receptor-binding affinities of both dual-targeting complexes for the human GRPR were determined by competition-binding assays at 4 °C in prostate adenocarcinoma PC3 cells against [^125^I-Tyr^4^]-BBN. The IC_50_ values were determined from the competitive receptor-binding curves represented in Figure S4. All BBN derivatives displaced the radioligand from the GRPR-binding sites in a dose-dependent way. The sub-nanomolar IC_50_ value of **Re-TPP-BBN** (0.38 ± 0.14 nM) was similar to the one obtained for the [Tyr^4^]-BBN reference peptide (0.56 ± 0.16 nM), the latest being very close to the nanomolar value reported in the literature (1.8 ± 0.2 nM) [32]. These results indicate that neither the introduction of the TPP moiety and complexation with Re reduced the binding affinity to the receptor. In contrast, a lower binding affinity was observed for the **Re-AO-BBN** complex (IC_50_ = 26.5 ± 6.8 nM) that could, in part, be responsible for the lowest level of cell internalization (Figure 3b).

### 2.3. Nuclear and Mitochondrial Uptake

Besides the ability of the radiocomplexes to be internalized by PC3 cells, their subcellular localization can influence the potential radiobiological effects—in particular, those induced by the AE emitted by ^99m^Tc. For the designed dual-targeted complexes, the cell nucleus and the mitochondria were identified as their putative radiosensitive subcellular targets. Thus, the nuclear and mitochondrial uptake of the different radiocomplexes under study were assessed using the appropriate commercial subcellular fractionation kits, as we described elsewhere [27,29].

**Figure 4:**
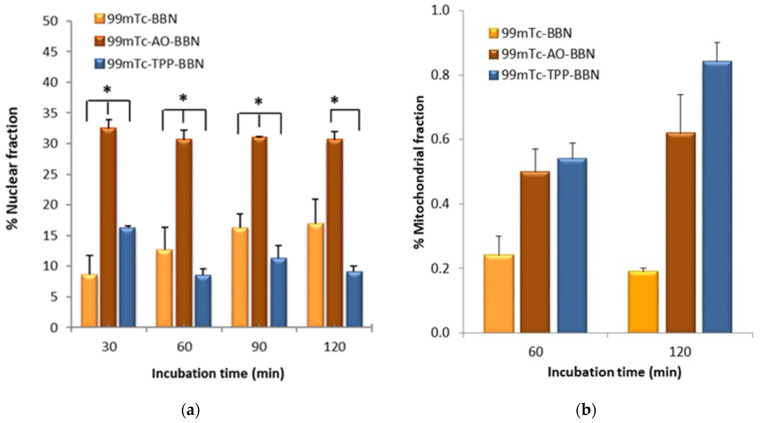
Nuclear uptake (**a**) and mitochondrial uptake (**b**) of **^99m^Tc-BBN**, **^99m^Tc-AO-BBN** and **^99m^Tc-TPP-BBN** in PC3 cells at 37 °C expressed as a percentage of the cell-associated activity (* *p* < 0.05).

As shown in Figure 4a, the AO-containing radiopeptide **^99m^Tc-AO-BBN** presented a fast and consistent nuclear uptake that spanned a very narrow range (30.5–32.4% of the radioactivity associated to the cells), significantly higher than that found for **^99m^Tc-BBN** (8.7–16.9%) and **^99m^Tc-TPP-BBN** (9.1–16.2%) (*p* < 0.05 when **^99m^Tc-AO-BBN** is compared with both **^99m^Tc-BBN** and **^99m^Tc-TPP-BBN** for all time points, except when **^99m^Tc-AO-BBN** is compared with **^99m^Tc-BBN** for 120 min).

The mitochondrial uptake values measured for **^99m^Tc-AO-BBN** (0.50 and 0.62% of the radioactivity associated with the cells for 60 and 120 min of incubation, respectively) are slightly lower than the corresponding values that we previously reported for **^99m^Tc-TPP-BBN** (0.54 and 0.84%) but higher than those found for **^99m^Tc-BBN** (0.24 and 0.19%) (Figure 4b) [27].

Fractionation assays can leave some organelles largely intact, namely the nucleus, while others such as the mitochondria can be damaged [5] with their contents released into other subcellular components. As a consequence, there is a cross-contamination between the mitochondrial and the cytosolic fractions based on the fractionation protocol, as we discussed elsewhere [27,33]. For these reasons, the mitochondrial uptake values measured have to be seen as relative values useful to rank the mitochondria-tropism of the radiocomplexes, which follows the order **^99m^Tc-TPP-BBN** > **^99m^Tc-AO-BBN** > **^99m^Tc-BBN**. This trend can be accounted for by the presence of the TPP pharmacophore, which has well-recognized mitochondrion tropic properties [34,35]. On the other hand, the AO group can act as a DNA intercalator with the ability to carry its compounds to the cell nucleus, as we previously showed based on fluorescence microscopy studies [30,36]. However, other authors also reported that some compounds carrying AO pharmacophores are able to accumulate in the mitochondria, with or without simultaneous localization in the nucleus [37,38,39]. The results obtained for **^99m^Tc-AO-BBN** seem to be in line with this possibility, as this radiocomplex showed some accumulation in the mitochondria, and the highest nuclear uptake that varied in the order **^99m^Tc-AO-BBN** > **^99m^Tc-BBN** > **^99m^Tc-TPP-BBN**.

### 2.4. Fluorescence Microscopy Studies

**Figure 5:**
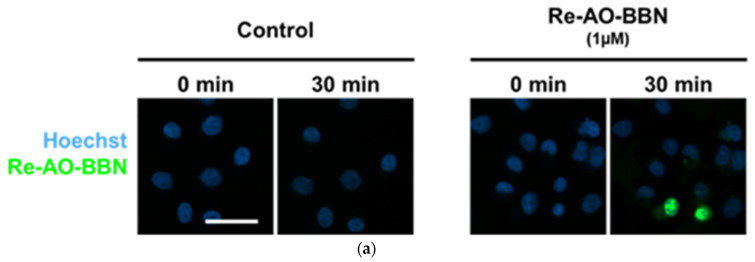

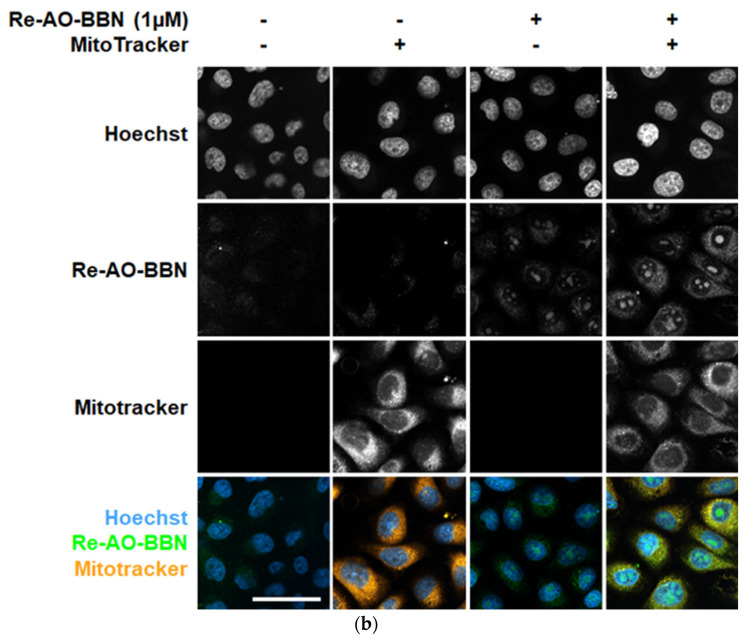
The cellular uptake and localization of **Re-AO-BBN** in PC3 evaluated by fluorescence microscopy. (**a**) Live cell uptake of **Re-AO-BBN** (1 µM). (**b**) Intracellular localization of **Re-AO-BBN** and MitoTracker. Confocal slices show nucleolar and cytoplasmic accumulation of **Re-AO-BBN**. Colocalization of **Re-AO-BBN** and MitoTracker fluorescence is quantified at Pearson’s r = 0.31. Mitochondrial morphology is not changed by the presence of **Re-AO-BBN**. Scale bar = 50 µm.

To evaluate the subcellular localization of the complex carrying the AO moiety and, in particular, the possibility of mitochondrial accumulation, we performed fluorescence microscopy studies with **Re-AO-BBN**, taking advantage of the fluorescent properties of the AO moiety. First, we performed a live cell wide field microscopy assay to assess the uptake kinetics of this complex in PC3 cells. As can be seen in Figure 5a, at 30 min, **Re-AO-BBN** was already accumulated in the nucleus, particularly in the nucleoli of the cells, while lower amounts were detected in the cytoplasm despite the cell-to-cell variations. This nuclear localization is in accordance with our previous studies [30,36] and with the high nuclear uptake of the radioactive congener **^99m^Tc-AO-BBN** (Figure 4a). 

We then performed the incubation of cells with **Re-AO-BBN** and Mitotracker Deep Red FM, a cell-permeable dye that stains the mitochondria, followed by fixation and confocal microscopy analysis (Figure 5b).

When the cells are fixed, we can observe that **Re-AO-BBN** shows a pronounced nuclear accumulation (as in the case of live cells) but, also, a diffuse cytoplasmic localization. Some colocalization is detected with the Mitotracker dye (Pearson’s Correlation Coefficient, PCC = 0.31), suggesting that, indeed, **Re-AO-BBN** is able to accumulate in the mitochondria of PC3 cells. This observation is consistent with the mitochondria uptake detected for **^99m^Tc-AO-BBN** (Figure 4b). However, unlike the extreme values, the meaning of the intermediate PCC values, as the ones obtained in our work, is often difficult to interpret. Dunn et al. outlined a consensus for PCC interpretation [40] in which colocalization was classified as high (PCC > 0.9), reasonably strong (PCC ~0.6), poor (PCC ~0.16) and not significant (PCC ~0.07). Horton et al. defined mitochondria localization mitochondria-penetrating peptides as very high (PCC ~0.5–0.6) or low (PCC ~0.2) [41], and similar values were reported for peptides with (PCC ~0.54–0.94) and without (PCC ~0.15) mitotropic features [42].

### 2.5. Evaluation of Radiobiological Effects

#### 2.5.1. Clonogenic Assays

**Figure 6:**
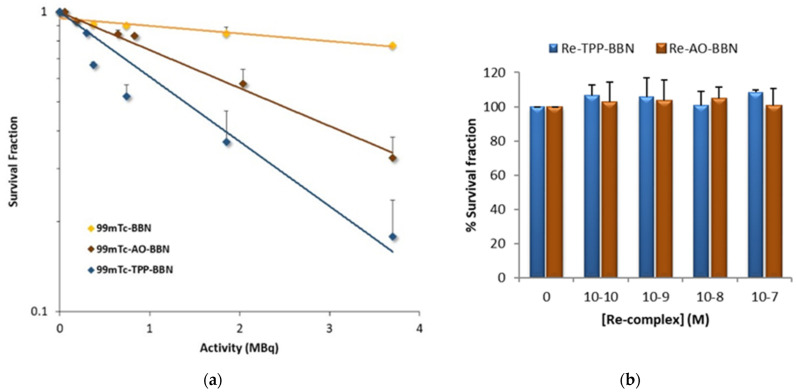
Survival fraction from clonogenic assay experiments. (a) PC3 cells were incubated with 0–3.7 MBq of HPLC-purified **^99m^Tc-BBN, ^99m^Tc-AO-BBN** and **^99m^Tc-TPP-BBN** for 24 h at 37 °C. The lethal dose (LD_50_) was >5, 2.92 and 1.39 MBq for **^99m^Tc-BBN, ^99m^Tc-AO-BBN** and **^99m^Tc-TPP-BBN**, respectively. (b) PC3 cells were incubated with **Re-AO-BBN** and **Re-TPP-BBN** complexes (0, 10^−7^, 10^−8^, 10^−9^ and 10^−10^ M) for 24 h at 37 °C. The data correspond to the mean ± SEM of 3 replicates.

We proceeded with the evaluation of the radiocytotoxicity of **^99m^Tc-AO-BBN** in PC3 cells, using the clonogenic assay upon exposure of the cells to increasing activities of the compound, as we previously described for **^99m^Tc-BBN** and **^99m^Tc-TPP-BBN** [27]. The clonogenic assay evaluates the ability of a single cell to grow into a colony, i.e., to undergo continuous proliferation, and is often used to study the effect of IR on the survival of cancer cells, either for EBRT or for TRT with medical radionuclides [43]. The results obtained for **^99m^Tc-AO-BBN** are presented in Figure 6a in comparison with **^99m^Tc-BBN** and **^99m^Tc-TPP-BBN** [27]. For all the tested activities, the dual-targeted **^99m^Tc-AO-BBN** induced a much stronger reduction in cell survival than the single-targeted **^99m^Tc-BBN,** although weaker than that caused by the other dual-targeted complex, **^99m^Tc-TPP-BBN**.

The clonogenic assays were performed with HPLC-purified radiocomplexes, in which the excess of the respective ligands was removed and did not contribute to the observed cytotoxicity. The total molar concentration of Tc (^99m^Tc and ^99^Tc) involved in the clonogenic assays was of the order of 10^−9^ M, even for the highest tested dose of 3.7 MBq, since the ^99m^TcO_4_^−^ used to prepare the radioconjugates was obtained by the elution of a ^99^Mo/^99m^Tc generator submitted 24 h before to a preventive elution. This preventive elution was performed to minimize the accumulation of reactive oxygen species (ROS) and ^99^Tc inside the generator column, which could introduce some bias on the observed radiobiological effects. 

We used the complexes **Re-AO-BBN** and **Re-TPP-BBN** as surrogates of the ^99m^Tc congeners and evaluated their influence in the survival of PC3 cells in the concentration range 10^−10^–10^−7^ M. Re complexes are commonly used as surrogates for ^99m^Tc congeners, as described above for the chemical identification of **^99m^Tc-AO-BBN** by HPLC in comparison with **Re-AO-BBN**, due to the physicochemical similarities of Tc and Re that form isostructural complexes with similar biological properties [44,45]. Even for the PC3 cells treated with the highest concentration of Re complexes (10^−7^ M), which is ca. 100-fold superior to that used in the clonogenic assays with the ^99m^Tc complexes, there was no effect on their survival when compared with the control (Figure 6b). Therefore, the cytotoxicity observed for **^99m^Tc-AO-BBN** and **^99m^Tc-TPP-BBN** is most probably the result of biological effects due to the radiation emitted by ^99m^Tc.

#### 2.5.2. γ-H2AX and Micronucleus Assays

**Figure 7:**
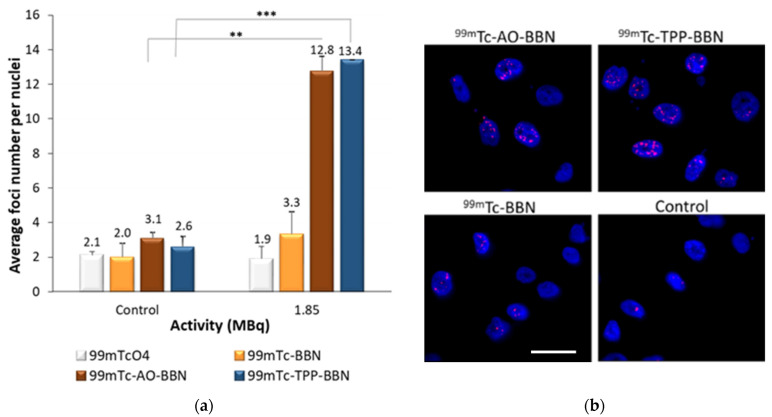
DNA damage induced by HPLC-purified **^99m^Tc-BBN**, **^99m^Tc-AO-BBN** and **^99m^Tc-TPP-BBN** and **^99m^TcO_4_** (1.85 MBq) in PC3 cells after 24 h of incubation at 37 °C. (**a**) The average number of foci per nuclei in PC3 cells, the data corresponding to the mean ± SEM of 3 replicates (** *p* < 0.01 and *** *p* < 0.005). (**b**) Fluorescence images of PC3 cells exposed to 1.85 MBq of ^99m^Tc complexes. Cells were immunostained for γ-H2AX, and DAPI was used to visualize the nuclei. Scale bar = 50 µm.

The phosphorylation of the Ser-139 residue of the histone variant H2AX, forming γ-H2AX, is an early cellular response to the induction of DSBs. The detection of this phosphorylation event has emerged as a highly specific and sensitive molecular marker for monitoring DNA damage [46]. The formation of a γ-H2AX foci can act as a molecular marker of cellular radiosensitivity in order to evaluate the efficacy of new therapeutic approaches and, also, to predict individual responses to IR even in clinical settings [47]. For this study, PC3 cells were incubated with 1.85 MBq of the different radiocomplexes under study for 24 h at 37 °C. ^99m^TcO_4_^−^, which is not internalized by the cells, was also evaluated under the same conditions, and basal control assays were also performed in parallel without any radioactive compounds. Several randomly selected images were collected for each condition, using a fluorescence microscope, which were then analyzed with Cellprofiler55^®^ [48]. The results, represented as the average number of foci per nuclei, are presented in Figure 7a. 

PC3 cells not exposed to the radioactive compounds presented a low average number of foci, very similar among the different assays, corresponding to the basal “background” DNA damage that naturally occurs. ^99m^TcO_4_^−^ did not cause any increase in the foci number when compared with the respective control. The **^99m^Tc-BBN** complex also did not significantly induce more DNA damage relative to the control. By contrast, the dual-targeted complexes **^99m^Tc-AO-BBN** and **^99m^Tc-TPP-BBN** led to a significant increase of the foci number in the PC3 cells, with the average number of foci increasing almost 4–5 times when compared to cells not exposed to the radiocomplexes. Altogether, these results show that the intracellular localization of the ^99m^Tc complexes plays a crucial role in the induction of DNA damage, which is more significant for the complexes preferentially located in the nucleus and/or the mitochondria.

**Figure 8:**
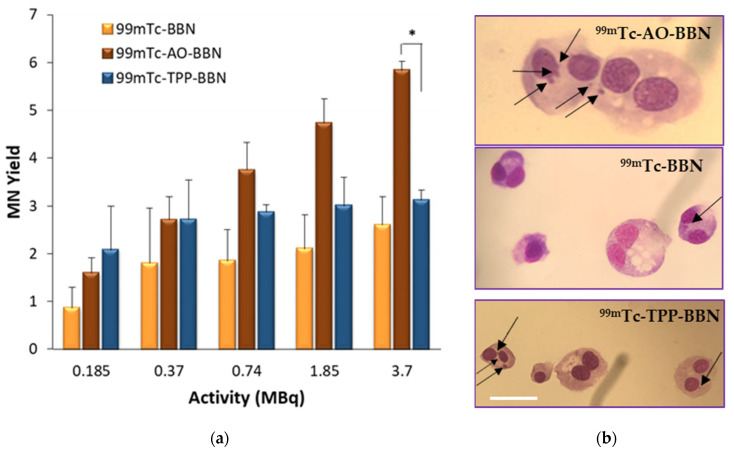
Micronucleus formation in PC3 cells exposed to the HPLC-purified **^99m^Tc-BBN**, **^99m^Tc-AO-BBN** and **^99m^Tc-TPP-BBN** (0.185–3.7 MBq) after 24 h of incubation with the compounds at 37 °C. (**a**) Activity-dependent MN yield normalized to the control. The data correspond to the mean ± SEM of 3 replicates (* *p* < 0.05). (**b**) Binucleated cells without and with MN observed at a x43-fold magnification. Scale bar = 35 µm.

Another biomarker that can be used to evaluate the effects of IR in cells is the formation of micronuclei. A micronucleus (MN) is an extranuclear body that forms during anaphase, as a result of unrepaired chromosome breaks, DNA misrepair or the formation of acentric chromosome fragments, namely upon the cell exposure to IR. The formation of micronuclei in the cells treated with complexes **^99m^Tc-BBN**, **^99m^Tc-AO-BBN** and **^99m^Tc-TPP-BBN** was evaluated by the cytokinesis block micronucleus assay (CBMN) [49]. The cells were exposed to the radiocomplexes for 24 h in the activity range 0.185–3.7 MBq, and the results were expressed as the MN yield corresponding to the distribution of the MN number per 1000 binucleated (BN) cells normalized to the control (Figure 8).

The dual-targeted complexes **^99m^Tc-AO-BBN** and **^99m^Tc-TPP-BBN** were more able to trigger the formation of the micronuclei compared with the single-targeted counterpart **^99m^Tc-BBN,** the respective MN yields appearing in the ranges 1.6–5.8, 2.1–3.1 and 0.9–2.6, respectively. For the highest tested activity (3.7 MBq), **^99m^Tc-AO-BBN** induced a significantly higher number of micronuclei when compared with **^99m^Tc-TPP-BBN** (*p* < 0.05). These results are contrary to those observed in the γ-H2AX assay, in which both compounds exhibited a similar behaviour. Compared with the γ-H2AX foci, MN formation can be seen as a late biological effect that is observed in cells that undergo cellular division and escape the DNA repair mechanisms. Thus, these trends seem to indicate that the DNA lesions induced by **^99m^Tc-AO-BBN** are less prone to be repaired than those induced by **^99m^Tc-TPP-BBN**.

DNA lesions, such as DSBs, can be produced by direct radiation hits like those resulting from DNA interactions with AEs emitted by ^99m^Tc [18]. However, despite their high LET, AEs can also contribute indirectly to the formation of DNA lesions through the formation of reactive oxygen species (ROS) generated in cells during oxidative stress [7,50,51,52,53]. As discussed above, **^99m^Tc-TPP-BBN** induced the formation of a significant number of γ-H2AX foci rather comparable to that induced by **^99m^Tc-AO-BBN**, even if **^99m^Tc-TPP-BBN** was the tested complex with the lowest uptake in the nucleus of the target PC3 cells. This result seems to indicate a substantial involvement of indirect processes in the DNA damage induced by **^99m^Tc-TPP-BBN**. **^99m^Tc-TPP-BBN** has led to a lower efficiency of MN formation when compared to **^99m^Tc-AO-BBN** but was able to affect the cellular proliferation of PC3 cells to a larger extent than the latter, probably due to its mitotropic nature.

#### 2.5.3. Effects on Mitochondrial Function

Mitochondria have a central role in cellular metabolism and are the principal player in the production of cellular energy through the formation of ATP by oxidative phosphorylation (OXPHOS) processes within the Krebs cycle and also being the main source of endogenous ROS. Consequently, mitochondria are strongly involved in the biological response of tumor cells to IR exposure, therefore considered radiosensitive organelles. In fact, similar to nuclear DNA, mitochondrial DNA (mtDNA) can also be damaged by direct radiation effects or by processes mediated by ROS. Most relevantly, mitochondrial DNA is not protected by histones and is less prone to undergo efficient repair mechanisms when compared to nuclear DNA [51,54]. As a consequence, the irradiation of mitochondria triggers radiation-induced cell signaling pathways such as apoptosis [51,55,56].

The subcellular localization studies showed that the complexes **^99m^Tc-TPP-BBN** and **^99m^Tc-BBN** have the highest and lowest mitochondrial uptakes, respectively. Most relevantly, the clonogenic assay results demonstrated that **^99m^Tc-TPP-BBN** reduces much more strongly the survival of PC3 cells than **^99m^Tc-BBN** for the same range of applied activities and in a dose-dependent manner. Thus, we selected these complexes to evaluate how the different mitochondrial uptakes influence the mitochondrial function of PC3 cells exposed to the compounds. For this study, we evaluated the effect of the exposure of the cells to **^99m^Tc-TPP-BBN** and **^99m^Tc-BBN** on the mtDNA copy number and on the ATP production.

**Figure 9:**
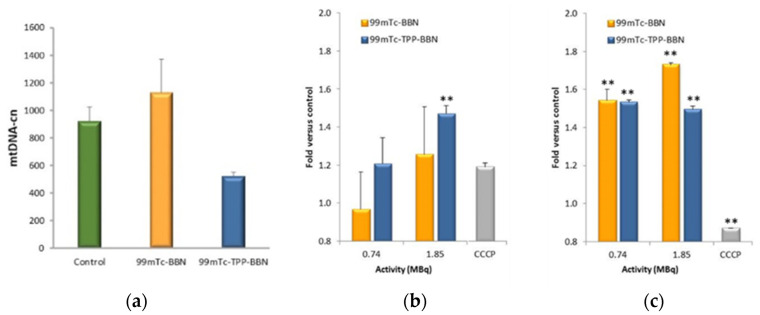
Evaluation of the mitochondrial effect. (**a**) Mitochondrial DNA copy number (mtDNA-cn) of PC3 cells after 2 h of incubation with 9.25 MBq/mL of **^99m^Tc-BBN** and **^99m^Tc-TPP-BBN** at 37 °C. Cells only with the regular medium were used as the control. (**b**) Membrane integrity and (**c**) ATP levels for PC3 cells treated for 2 h at 37 °C with 0.74 and 1.85 MBq of **^99m^Tc-BBN** and **^99m^Tc-TPP-BBN**. Cells incubated with the assay medium (HBSS supplemented with galactose and 1% BSA) were used as a basal control, and cells incubated with CCCP (10 μM) as a positive control for mitochondrial inhibition. The data are represented as the fold versus the basal control. The data correspond to the mean ± SEM of 3 replicates (** *p* < 0.01 versus the control).

To assess the effect on the mtDNA copy number, PC3 cells were incubated with 9.25 MBq/mL of each complex for 2 h at 37 °C. The applied activity used in this study was the same as that used in the γ-H2AX assay, being the same order of magnitude as the LD_50_ obtained for **^99m^Tc-TPP-BBN** in the clonogenic assay. After exposure to the compounds, the total DNA was extracted based on a commercial kit, and the mtDNA copy number was determined using a qPCR technique, as detailed in the experimental section. The obtained results, relative to the control cells incubated only with the cell medium, are presented in Figure 9a. It was found that the mtDNA copy number of the control cells and PC3 cells treated with **^99m^Tc-BBN** were not statistically different. On the contrary, **^99m^Tc-TPP-BBN** led to an approximate 50% reduction of the mtDNA content when compared to the control cells, indicating that this mitotropic complex might have the ability to induce mitochondrial dysfunction with an impact on the mtDNA content.

To the best of our knowledge, no studies have been previously reported to evaluate the effect of radionuclides internalized by tumor cells on their mtDNA copy number. In contrast with the results obtained for **^99m^Tc-TPP-BBN**, the external irradiation of cells with IR (e.g., X-ray or α-particles) often leads to an increased mtDNA copy number, eventually compensating the poor repair capability of mtDNA and acting as a self-protective mechanism of tumor cells to prevent apoptosis [56,57,58]. Interestingly, cytotoxic drugs that are able to accumulate in the mitochondria and bind to mtDNA, such as the classical chemotherapeutic drugs cisplatin and doxorubicin or a Ru^II^–Pt^II^ bimetallic complex, inhibit mtDNA synthesis and cause its depletion [59,60,61], as we observed for **^99m^Tc-TPP-BBN**. It is possible to hypothesize that the localization of **^99m^Tc-TPP-BBN** in the mitochondria could favor the occurrence of direct mtDNA damage induced by the AEs emitted by the radionuclide.

Finally, we studied the metabolic activity of PC3 cells by measuring their ATP production following the treatment of the cells with different activities of **^99m^Tc-TPP-BBN** and **^99m^Tc-BBN** for 2 h at 37 °C. The study was performed using the commercially available kit “Mitochondrial ToxGlo™ Assay” that quantifies biomarkers associated with changes in the cell membrane integrity and cellular ATP levels. It first measures the cell membrane integrity with a fluorescent probe that cannot cross the membrane of viable cells. An increase in fluorescence of the cells exposed to a toxic agent in comparison with a control reflects the loss of membrane integrity and cytotoxicity. Next, cellular ATP production is measured in a luminescent assay. By using a galactose-containing medium (versus glucose), the oxidative phosphorylation pathway to generate ATP is favored over glycolysis, and this leads to the best conditions to evaluate the effects on mitochondrial ATP production. The combination of the two sets of data (cell death and ATP production) can give hints about the involvement of mitochondrial dysfunction or non-mitochondrial-associated cytotoxic mechanisms. This study was also conducted for cyanide-m-chlorophenylhydrazone (CCCP) [35], which is a mitochondria toxin that does not compromise the cell viability at the concentration tested. The results are presented in Figure 9.

As expected, the exposure of PC3 cells to CCCP did not compromise their viability (Figure 9b) but significantly reduced the ATP production when compared to the control cells, as the compound acts as an OXPHOS uncoupler (Figure 9c). **^99m^Tc-TPP-BBN** and **^99m^Tc-BBN** caused similar effects in the metabolism of PC3 cells, i.e., a significant increase of the ATP levels (Figure 9c).

In which concerns the membrane integrity, the effects were more pronounced for **^99m^Tc-TPP-BBN**, which caused a significant loss of membrane integrity at the highest activity tested (*p* < 0.01 vs control), while the effect of **^99m^Tc-BBN** was not significant (Figure 9b). This result indicates that **^99m^Tc-TPP-BBN** seems to be more effective in the induction of cell death than **^99m^Tc-BBN**, which is in accordance with the greater decrease in survival observed in the clonogenic assay (Figure 6a).

Unlike **^99m^Tc-BBN**, mitotropic **^99m^Tc-TPP-BBN** decreases the expression of the mtDNA of PC3 cells in the same way as described for different cytotoxic drugs, possibly due to localized mitochondria-targeted effects involving the AEs emitted by ^99m^Tc. However, the decrease in mtDNA copy number did not imply a reduction of the ATP production in the mitochondria. On the contrary, there was an augmented ATP production upon exposure of the cells to the compound, as was also observed for the cells treated with **^99m^Tc-BBN**.

Other authors reported a similar effect following the external irradiation of tumor cells with X-rays, which was attributed to OXPHOS enhancement as a response to cellular stress induced by the radiation [62]. By its nature, external X-ray irradiation does not allow the selective irradiation of mitochondria, such as **^99m^Tc-BBN** due to its subcellular distribution. The enhanced ATP production observed for the PC3 cells treated with **^99m^Tc-BBN** and **^99m^Tc-TPP-BBN** certainly reflected the overall irradiation of the cells by the AEs, X-ray and γ-photons emitted by the complexes, and not only the mitochondria-targeted effects, and might be interpreted as an initial sign of apoptosis, which is an energetically demanding cell death mechanism. In summary, **^99m^Tc-TPP-BBN** reduced the mtDNA copy number of PC3 cells, but apparently, the cells still retained a high mitochondrial ATP production after exposure to the compound. To better understand this result, further studies are needed. Namely, these studies should involve the evaluation of the influence of different times of exposure to **^99m^Tc-TPP-BBN** on the relationship between the mtDNA copy number and ATP production, as well as different time points after the incubation period. Furthermore, additional studies should be extended to other tumoral cell lines, as the radiobiological effects might be cell line-dependent. This could include other human tumoral cells with different levels of GRPR expression and normal cells from the same tissue. However, these more detailed studies are out of the scope of the present contribution.

## 3. Materials and Methods

Unless otherwise stated, all chemicals and solvents were of reagent grade and used without further purification. Compound **1**, ethyl 4-((2-(tert-butoxycarbonylamino)ethyl)(2-(4-(2-(2,5-dioxopyrrolidinyloxy)-2-oxoethyl)-3,5-dimethylpyrazol)ethyl)amino) butanoate, was prepared as described in the literature [63]. Compound **2**, 3,6-bis(dimethylamino)-10-(3-(1,3-dioxoisoindolin-2-yl)-propyl)acridinium iodide (**2**), was prepared according to the published methods [64]. The starting material *fac*-[Re(H_2_O)_3_(CO)_3_]Br was synthesized according to the literature [65]. The G3-BBN [7-14] peptide was synthesized by a standard Fmoc strategy, as described in the literature [35]. The G3-BBN [7-14] derivatives **Pz-BBN, TPP-BBN, Re-BBN** and **Re-TPP-BBN** were prepared as previously described [27,30]. Na[^99m^TcO_4_] was eluted from a commercial ^99^Mo/^99m^Tc generator using a 0.9% saline solution. The radioactive precursor *fac*-[^99m^Tc(CO)_3_(H_2_O)_3_]^+^ was prepared as we described earlier [18,30].

^1^H-, ^13^C- and ^31^P-NMR spectra were recorded on a Bruker Avance III 400 MHz or 300 MHz spectrometers. The chemical shifts (δ) are given in ppm and were referenced to the residual solvent resonances relative to tetramethylsilane (SiMe_4_). Coupling constants (*J*) are given in Hz.

Mass spectra were acquired in an electrospray ionization/quadrupole ion trap (ESI/QITMS) Bruker HCT mass spectrometer (Bruker, Billerica, MA, USA). Samples were injected in mixtures of water:acetonitrile or water:methanol and injected at a flow rate of 150 µL·h^−1^.

Column chromatography was performed with silica gel 60 (Merck). HPLC (Perkin Elmer, Waltham, MA, USA) analysis of the Re and ^99m^Tc complexes was performed on a Perkin–Elmer LC pump 200 coupled to a LC 290 tunable UV–Vis detector and to a Berthold LB-509 radiometric detector, using an analytic Macherey-Nagel C18 reversed-phase column (Nucleosil 100–10, 250 × 4 mm) with a flow rate of 1 mL/min. HPLC solvents consisted of 0.1% CF_3_COOH in H_2_O (eluent A) and 0.1% CF_3_COOH in acetonitrile (eluent B), and two different gradients were used: HPLC (method I): the gradient was: t = 0–25 min, 10–90% eluent B; 25–27 min,10–100% eluent B; 27–30 min, 100% eluent B; 30–31 min, 100–10% eluent B; 31–35 min, 10% eluent B. HPLC (method II): the gradient was: t = 0–3 min, 0% eluent B; 3–3.1 min, 0–25% eluent B; 3.1–9 min, 25% eluent B; 9–9.1 min, 25–34% eluent B; 9.1–25.1 min, 34–54% eluent B; 25.1–26 min, 54-0% eluent B; 26–30 min, 0% eluent B.

### 3.1. Synthesis of Acridine Orange Derivatives

#### 3.1.1. Ethyl 4-((2-(4-(2-(10-4-(Propylamino)-3,6-bis(dimethylamino)acridinium)-2-oxoethyl)-3,5-dimethylpyrazol)ethyl) (2-(Tert-butoxycarbonylamino) thyl)amino) Butanoate (3)

DIPEA (0.355 mmol) was added to a solution of 3,6-bis(dimethylamino)-10-(3-(1,3-dioxoisoindolin-2-yl)-propyl)acridinium iodide (0.160 g, 0.355 mmol) in dry DMF (5 mL), and the mixture was stirred for 1 h. Then, compound **1**, ethyl 4-((2-(tert-butoxycarbonylamino)ethyl)(2-(4-(2-(2,5-dioxopyrrolidinyloxy)-2-oxoethyl)-3,5-dimethylpyrazol)ethyl)amino) butanoate (0.220 g, 0.40 mmol), in DMF (5 mL) was added, and the mixture was stirred for 3 days at room temperature. Then, the solvent was evaporated, and the residue was purified with silica gel column chromatography (eluent: EtOH, 100→50/CHCl_3_, 0→50/NH_3_, 5→20. Rf (NH_3_, 0.1 mL/EtOH, 7 mL/CHCl_3_, 3 mL) = 0.14. Yield: 0.103 g; 30 %. 

^1^H NMR (300 MHz, CD_3_OD): δ (ppm) 1.21 (t, 3H, CH3); 1.28 (s br, 2H, CH_2_); 1.41 (s, 9H, 3CH_3_, BOC); 1.59 (m, 2H, CH_2_); 2.24–2.15 (m, 6H + 4H, CH_3_/CH_2_); 2.43 (t, 3H, CH_3_); 2.49 (t, 3H, CH_3_); 2.74 (t, 3H, CH_3_); 3.01 (m, 2H, CH_2_); 3.32 (s, 12H, CH_3_); 3.50 (t, 2H, CH_2_); 4.06 (m, 4H, CH_2_); 4.69 (t, 2H, CH_2_); 6.66 (s, 2H, 2CH-Ar); 7,27 (dd, 2H, 2CH-Ar); 7.90 (d, 2H, 2CH-Ar); 8,64 (s, H, CH-Ar).

^13^C NMR (75 MHz, CD_3_OD): δ (ppm) 9.96 (CH_3_), 11.90 (CH_3_), 14.59 (CH_3_-Et), 23.68, 27.02, 28.80 (3CH_3_-BOC), 30.68, 31.91, 38.32, 29.50, 40.87 (4CH_3_), 46.53, 54.52, 54.81, 54.95, 61.37 (CH_2_-Et), 79.97 (C-BOC), 93.45 (2CH-Ar), 111.20 (C(4)pz), 115.54 (2CH-Ar), 118.50 (C-Ar), 134.42 (2CH-Ar), 139.65 (C3/5-pz), 144.08 (CH-Ar), 144.37 (C-Ar), 147.65 (C3/5-pz), 157.45 (C=O), 174.35 (C=O), 175.37 (C=O). 

ESI-MS (+) *m/z* calcd for [C_42_H_63_N_8_O_5_]^+^ = 759.49; found 759.5.

HPLC (method I): Rt = 17.2 min.

#### 3.1.2. 4-((2-(4-(2-(10-4-(Propylamino)-3,6-bis(dimethylamino)acridinium)-2-oxoethyl)-3,5-dimethylpyrazol)ethyl) (2-(Tert-butoxycarbonylamino) ethyl)amino) Butanoic Acid (4)

To a solution of **1** (0.060g; 0.068 mmol) in distilled H_2_O (3 mL) were added 0.5 mL of 1 M NaOH aqueous solution. This mixture was heated at 75 °C overnight. The next day, a solution of 3 M aqueous HCl was added dropwise to the mixture until reaching a final pH of about 7. The product was extracted with chloroform, dried with MgSO_4_ and the solvent was removed under vacuum. The compound was recovered after purification with Sep Pak OASIS MAX cartridges (Waters Co, 6 cc/150 mg 30 μm, Part No. 186000369) preconditioned according to the manufacturer’s protocol using a MeOH–H_2_O gradient. After washing with 5 mL of 5% NH_4_OH in H_2_O, the product was eluted with 5 mL of methanol. Yield: 32.5 mg; 56.1%. 

^1^H NMR (300 MHz, CD_3_OD): δ (ppm) 1.37 (s, 9H, CH_3_); 1.63 (m br, 2H, CH_2_); 2.13–2.22 (m, 10H, 2CH_3_ + 2CH_2_); 2.53–2.63 (m, 4H, CH_2_); 2.89 (t, 2H, CH_2_, J = 6.0); 3.05 (m, 2H, CH_2_); 3.31 (m, 14H, CH_2_ + CH_3_); 3.49 (t, 2H, CH_2_, J = 7.26); 4.07 (t, 2H, CH_2_, J = 5.90); 7.72–4.63 (m, 2H, CH_2_); 6.65 (s, 2H, CH-Ar); 7.25 (dd, 2H, CH-Ar, J = 9.28, J = 1.98); 7.88 (d, 2H, CH-Ar, J = 9.34); 8,63 (s, H, CH-Ar).

^13^C NMR (75 MHz, CD_3_OD): δ (ppm) 9.96 (CH_3_), 11.90 (CH_3_), 23.84 (CH_2_), 27.02 (CH_2_), 28.77 (3CH_3_, BOC), 31.98 (CH_2_), 34.82 (CH_2_), 38.35 (CH_2_), 38.96 (CH_2_), 40.89 (4CH_3_), 46.55 (CH_2_), 47.53 (CH_2_), 48.15 (CH_2_), 54.76 (CH_2_), 54.83 (CH_2_), 55.44 (CH_2_), 80.07 (C-BOC), 93.49 (2CH-Ar), 111.19 (C(4) pz), 115.54 (2CH-Ar), 118.50 (4C-Ar), 134.41 (2CH-Ar), 139.89 (C3/C5 pz), 144.08 (2C-Ar), 144.35 (CH-Ar), 147.93 (C3/C5 pz), 157.46 (2CH-Ar), 158.28 (C=O, BOC), 174.43 (C=O), 179.72 (C=O).

ESI-MS (+) *m/z* calcd for [C_40_H_59_N_8_O_5_]^+^ = 731.46; found: 731.8. 

HPLC (method I): Rt = 16.5 min.

#### 3.1.3. 4-((2-(4-(2-(10-(4-Butylamino)-3,6-bis(dimethylamino)acridinium)-2-oxoethyl)-3,5-dimethylpyrazol)ethyl)(2-aminoethyl)amino) Butanoic Acid (L1)

A solution of **4** (12.1 mg; 0.0141 mmol) and trifluoroacetic acid (1 mL) in 1 mL of dichloromethane was reacted at room temperature over five hours. The product obtained was pure after evaporating the solvent under reduced pressure. Yield: 10.5 mg; 98%.

^1^H NMR (300 MHz, CD_3_OD): δ (ppm) 1.76–1.62 (m, 2H, CH_2_); 2.25–2.09 (m, 10H, 2CH_3_ + 2CH_2_); 2.78 (t, 2H, CH_2_, *J* = 6.87); 3.05–3.01 (m, 2H, CH_2_); 3.14–3.09 (m, 6H, CH_2_); 3.31 (m, 14H, CH_2_ + CH_3_); 4.48 (t, 2H, CH_2_, *J* = 7.25); 4.20 (t, 2H, CH_2_, *J* = 5.80); 4.69 (m, 2H, CH_2_); 6.66 (s, 2H, CH-Ar); 7.25 (dd, 2H, CH-Ar, *J* = 9.31, *J* = 1.99); 7.88 (d, 2H, CH-Ar_,_ *J* = 9.36); 8,63 (s, H, CH-Ar).

^13^C NMR (75 MHz, CD_3_OD): δ (ppm) 9.59 (CH_3_), 11.74 (CH_3_), 21.94, 27.06, 30.76, 31.51, 37.60, 38.33, 40.86 (4CH_3_), 46.12, 46.47, 51.77, 54.00, 54.35, 93.48 (2CH-Ar), 112.02 (C(4)pz), 115.56 (2CH-Ar), 118.53 (2C-Ar), 134.44 (2CH-Ar), 140.04 (C3/5pz), 144.40 (CH-Ar), 148.11 (C3/5pz), 157.49 (C-Ar), 174.13 (C=O ), 176.82 (C=O).

ESI-MS (+) *m/z* calcd for [C_35_H_51_N_8_O_3_^+^] = 631.41; found: 632.5. calcd for [M+H]^2+^ = 316.7; found: 317.1.

HPLC (method II): Rt = 15.4 min.

#### 3.1.4. Synthesis of the Re(I) Complex

*fac*-[Re(CO)_3_(*k*^3^-L1)]^2+^(CF_3_CO_2_Na) (**Re-AO-COOH**) was obtained by a reaction of [Re(H_2_O)_3_(CO)_3_]Br with an equimolar amount of **L1** in refluxing H_2_O (12 h). The solvent was removed under reduced pressure, and the product was extracted with methanol to give **Re-AO-COOH**. Yield: 18.7 mg; 90%. 

^1^H NMR (300 MHz, CD_3_OD): δ (ppm) 1.93–2.05 (m, 1H, CH_2_), 2.07–2.27 (m, 2H, CH_2_), 2.31 (s, 3H, CH_3_), 2.39 (s, 3H, CH_3_), 2.42 (m, 2H, CH_2_), 2.58–2.68 (m, 1H, CH_2_), 2.71–2.78 (m, 1H, CH), 2.87 (m, 2H, CH_2_), 3.09–3.19 (m, 2H, CH_2_), 3.32 (m, CH_3_, 12H), 3.42 (s, CH_2_, 2H), 3.52 (m, 4H, CH_2_), 3.68 (m, 1H, CH), 4.04 (s br, 1H, NH), 4.22 (m, 1H, CH), 4.54 (m, 1H, CH), 4.73 (t, 2H, CH_2_), 5.51 (q br, 1H, NH), 6.68 (s, 2H, CH-Ar); 7.26 (dd, 2H, CH-Ar, *J* = 9.29, *J* = 1.80); 7.89 (d, 2H, CH-Ar, *J* = 9.34); 8.64 (s, H, CH-Ar).

^13^C NMR (75 MHz, MeOD) δ (ppm) 194.95 (C≡O), 194.88 (C≡O), 193.82 (C≡O), 176.16 (C=O), 173.17 (C=O), 157.47 (2C-Ar), 153.97 (C3/C5 pz), 144.38 (CH-Ar), 144.10 (2C-Ar), 144.03 (C3/C5 pz), 134.43 (2CH-Ar), 118.51 (2C-Ar), 115.54 (2CH-Ar), 114.06 (C(4)pz), 93.53 (2CH-Ar), 66.94 (CH_2_), 62.40 (CH_2_), 53.69 (CH_2_), 48.43 (CH_2_ under residual MeOH, as demonstrated by the HSQC experiment), 46.42 (CH_2_), 43.60 (CH_2_), 40.91 (4CH_3_), 38.43 (CH_2_), 31.51 (CH_2_), 31.34 (CH_2_), 27.01 (CH_2_), 20.49 (CH_2_), 14.71 (CH_3_) and 10.37 (CH_3_).

ESI-MS (+) calcd for [C_38_H_51_N_8_O_6_Re + CF_3_COONa]^2+^ = 519.16; found: 519.9.

HPLC (method II): Rt = 20.4 min.

#### 3.1.5. Synthesis of the Peptide Conjugate (AO-Pz-BBN)

Compound **4** was coupled to the *N*-terminal of the G3-BBN [7-14] peptide (2.5 equiv) in DMF, in the presence of O-benzotriazole-*N*,*N*,*N*′,*N*′-tetramethyluronium hexafluorophosphate (HBTU: 2.5 equiv) and DIPEA (4.6 equiv). The reaction was run for 4 h at room temperature, under a nitrogen atmosphere. After this time, the solvent was evaporated to dryness and the residue was treated with a mixture of TFA and dichloromethane (1:1) for 2 h at room temperature, under a nitrogen atmosphere, to remove the Boc protecting group from the terminal amine of the pyrazole-diamine framework. Thereafter, the solution was concentrated under a nitrogen stream and precipitated in Et_2_O, after standing in the freezer for 1 h. The resulting precipitate was washed two times with cold Et_2_O, dried and purified by RP-HPLC to yield the peptide conjugate **AO-Pz-BBN**, which was characterized by analytical HPLC and ESI-MS.

ESI-MS m/z calcd for [C_84_H_123_N_24_O_14_S+2H]^3+^ = 575.65; found 575.7. 

HPLC (method II): Rt = 17.5 min. 

#### 3.1.6. Synthesis of the Re(I) Conjugate (Re-AO-BBN)

The complex **Re-AO-COOH** was coupled to the *N*-terminal of the G3-BBN [7-14] peptide (1.1 equiv) using HBTU (1.2 equiv) and DIPEA (4.6 equiv) in the DMF solution and reacted for 3 h at room temperature, under a nitrogen atmosphere. The resulting mixture was dried under vacuum, purified by RP-HPLC and the recovered conjugate characterized by ESI-MS.

ESI-MS *m/z* calcd for [C_87_H_123_N_24_O_17_SRe + H]^3+^ = 665.29; found [M+H]^3+^ 665.7; calcd for [C_87_H_123_N_24_O_17_SRe]^2+^ = 997.44; found [M]^2+^ 997.9.

HPLC ((method II): Rt = 24.4 min.

### 3.2. Synthesis and In Vitro Evaluation of ^99m^Tc(I) Complexes

#### 3.2.1. Radiolabeling Procedure

The radiocomplexes **^99m^Tc-BBN** and **^99m^Tc-TPP-BBN** were obtained by the reactions of the respective ligands with the organometallic precursor *fac*-[^99m^Tc(CO)_3_(H_2_O)_3_]^+^, as we described elsewhere [27]; **^99m^Tc-AO-BBN** was obtained using the same procedure: to a nitrogen-purged vial containing the **AO-Pz-BBN** ligand (using concentrations as low as 10^−4^ M) was added the organometallic precursor *fac*-[^99m^Tc(CO)_3_(H_2_O)_3_]^+^ (2–5 mCi) in aqueous solution at pH 7. The reaction mixture was then heated at 100 °C for 30 min to give the desired ^99m^Tc complex (**^99m^Tc-AO-BBN**). After cooling to room temperature, the radiochemical purity (RCP) was determined by RP-HPLC analysis. **^99m^Tc-AO-BBN** was purified by HPLC to remove the excess of the respective ligand. The purified ^99m^Tc complex was collected in 0.1 M PBS pH 7.4 containing 0.2% of BSA to avoid adsorption to the vials and the organic solvent was removed under a nitrogen stream. The chemical identity of **^99m^Tc-AO-BBN** was established by comparison of its HPLC profile with that of the corresponding rhenium complex.

**^99m^Tc-AO-BBN**: HPLC: Rt = 25.0 min (γ detection; method II); RCP > 98% (after purification by HPLC).

#### 3.2.2. In Vitro Stability Studies

Radiochemical stability of **^99m^Tc-AO-BBN** was assessed by RP-HPLC analysis at several time points (2 h, 4 h and 24 h) following the incubation of the compound in PBS pH 7.4 and in DMEM cell culture medium at 37 °C, respectively.

#### 3.2.3. Lipophilicity Determination

The lipophilicity of **^99m^Tc-AO-BBN** was evaluated by the “shake flask” method [31]. Briefly, 25 µL of the radiocomplex solution were added to a mixture of n-octanol (1 mL) and PBS pH = 7.4 (1 mL), previously saturated in each other by stirring the mixture. This mixture was vortexed and centrifuged (5000 rpm, 10 min, room temperature) to allow phase separation. Aliquots of 50 µL of both octanol and PBS were counted in a gamma counter. The partition coefficient (P_o/w_) was calculated by dividing the counts in the octanol phase by those in the buffer, and the results were expressed as Log P_o/w_. Log P_o/w_= −0.84 (**^99m^Tc-AO-BBN**).

### 3.3. Cellular Studies

#### 3.3.1. Cell Culture

PC3 human prostate cancer cells (ECACC 90112714, England, UK) were grown in DMEM containing GlutaMax supplemented with 10% heat-inactivated fetal bovine serum and 1% penicillin/streptomycin antibiotic solution (all from Gibco, Thermo Fisher Scientific, Waltham, MA, USA) in a humidified atmosphere of 95% air and 5% CO_2_ at 37 °C (Heraeus, Hanau, Germany).

#### 3.3.2. Internalization and Cellular Uptake

Time-dependent accumulation of **^99m^Tc-AO-BBN** in PC3 cells was studied, as we have previously described for **^99m^Tc-BBN** and **^99m^Tc-TPP-BBN** [27]. PC3 cells were seeded at a density of 0.125 million per well in 24-well plates and allowed to attach overnight. The cells were incubated at 37 °C for a period of 5 min to 3 h with about 7.4 kBq (0.2 µCi) of the HPLC-purified radiocompound in 0.5 mL of culture medium. After each incubation time, the unbound radiocomplex was removed and the cells washed with ice-cold DMEM medium. Cell surface-bound radiocompound was removed by two steps of acid wash (50 mM glycine-HCl/100 mM NaCl, pH 2.8) at room temperature for 4 min. The pH was neutralized with cold PBS with 0.2% BSA, and subsequently, the cells were lysed with 1 M NaOH for 10 min at 37 °C to determine the internalized radiocompound. The activity in both cell surface-bound and internalized fractions was measured using a gamma counter (LB 2111, Berthold) and is reported as a proportion to the total applied radioactivity, together representing the cellular uptake of the radiocomplex. Assays for each time point were performed in quadruplicate, and data were presented as average ± SEM of typically three independent experiments.

For assessing the specific GRPR-mediated cellular uptake and internalization of **^99m^Tc-AO-BBN**, a similar study was performed in which the radiocomplex was incubated for 0.5 h and 1 h, with or without the potent GRPR agonist [Tyr^4^] Bombesin (0.25 µg/0.5 mL/well).

#### 3.3.3. Competitive Radioligand Binding Assay

The in vitro cell-binding assays were performed with the prostate carcinoma cell line PC3. Briefly, cells were seeded in 24-well plates (150,000 cells per well) and allowed to attach overnight. On the day of the experiment, cells were rinsed with ice-cold binding assay medium (RPMI 1640 medium supplemented with 1% (*v/v*) FBS, 25 mM HEPES and 1% (*w/v*) BSA). Competition was conducted by incubation of [^125^I-Tyr^4^]BBN (15,000 cpm, 0.2 mL, PerkinElmer, Inc., Boston, MA, USA) in the presence of increasing concentrations (10^−13^ to 10^−5^ M) of BBN-bearing Re-complexes in the binding buffer (0.1 mL, total volume per well 0.3 mL) for 90 min at 4 °C. The competition was interrupted by discarding supernatants and washing the cells twice with ice-cold PBS with 0.2% BSA. Cells were then lysed with 1 M NaOH treatment (2 × 0.4 mL, 10 min at 37 °C). Lysates were collected and counted for their radioactivity content in an automated γ–counter (HIDEX AMG, Hidex, Turku, Finland). IC_50_ values (concentration of competitor required to inhibit 50% of the radioligand binding) were calculated by nonlinear regression according to a one-site model using GraphPad PRISM 5 software (San Diego, CA, USA) and are the average of three independent experiments. Nonspecific binding was defined as the amount of activity bound in the presence of 10^−5^ M of the respective BBN-bearing Re-complex or [Tyr^4^]BBN.

#### 3.3.4. Nuclear Uptake

PC3 cells were seeded at a density of 50,000 cells per well in a 24-well plate and allowed to attach overnight. The cells were incubated with different activities (0.185–7.4 MBq) of HPLC-purified **^99m^Tc-BBN**, **^99m^Tc-TPP-BBN** and **^99m^Tc-AO-BBN** in 0.5 mL of culture medium for 24 h at 37 °C. Incubation was terminated by washing the cells with ice-cold PBS. Cells were detached from the plates using trypsin/EDTA solution (Gibco-Invitrogen). The unbound radioactive compound was removed by centrifugation of the cell suspension, followed by washing the cellular pellet with ice-cold PBS with 0.2% BSA. The activity of cellular pellet was measured, using a gamma counter, to quantify the total cellular uptake of the radiocompound. The pellet was then resuspended in 2 mL of ice-cold cell lysis buffer (10 mM Tris, 1.5 mM MgCl_2_ and 140 mM NaCl) containing 0.1% of IGEPAL-ca 630 (Sigma) and incubated on ice for 10 min to disrupt the cell membrane. After the lysis, the suspension was centrifuged at 1300× *g* for 2 min at 4 °C, the supernatant (cytoplasm) was separated from the pellet (nuclei), and the activity in both fractions measured. The nuclear uptake was expressed in a percentage of internalized activity.

#### 3.3.5. Mitochondrial Uptake

The mitochondrial uptake of **^99m^Tc-AO-BBN** was assessed based on compartmental fractionation studies in PC3 cells, as we previously described for **^99m^Tc-BBN** and **^99m^Tc-TPP-BBN** [27]. Adherent and confluent cells (T75 culture flask) were incubated with about 7.4 MBq (100 µCi) of the HPLC purified radiocompounds in 2 mL of culture medium for 1 h and 2 h at 37 °C and 5% CO_2_. The harvested cell suspension with 10–20 × 10^6^ cells was split into 3 fractions (3 replicates, 2 mL each), centrifuged at 850× *g* for 2 min at 4 °C (Centrifuge 5804R, Eppendorf) and cells washed twice with cold PBS to remove the unbound radiocomplex and the activity of whole-cell fraction was measured (cellular uptake determination). To obtain the mitochondrial fraction, the cells were treated with the “Mitochondria Isolation Kit for Cultured cells” (Thermo Scientific, Walthman, MA, USA) according to the manufacturer’s protocol. Briefly, the pellet (one-third of total pellet) was resuspended with reagent A (0.8 mL/2 × 10^7^ cells) supplemented with a cocktail of protease inhibitors (Roche, Basel, Switzerland), vortexed at medium speed for 5 s and incubated on ice for exactly 2 min. Reagent B (10 µL/2 × 10^7^ cells) was added and the samples were vortexed at maximum speed for 5 s, incubated on ice, and vortexed every minute for 5 min. Then, reagent C (0.8 mL/2 × 10^7^ cells), supplemented with a cocktail of protease inhibitors, was added, and the cells suspension centrifuged for 10 min at 700× *g* at 4 °C (Centrifuge 5417R, Eppendorf, Hamburg, Germany). The nucleus isolation steps were monitored by trypan blue staining under a phase contrast microscope. The resulting pellet corresponds to the nuclear fraction. The supernatant, transferred to a new tube, was centrifuged for 15 min at 3000× *g* at 4 °C to remove the lysosomal and peroxisomal contaminants. The pellet, containing the isolated mitochondria, was again treated with reagent C (0.5 mL/2 × 10^7^ cells) and centrifuged for 5 min at 12,000× *g*. The activity in all fractions collected were directly counted in a dose calibrator (Berthold, LB2111, Bad Wildbad, BW, Germany).

#### 3.3.6. Cellular Uptake by Fluorescence Microscopy

PC3 cells seeded at a density of 5000 cells per well in microscopy-grade 96 well plate (Greiner, 655090, Frickenhausen, Germany) and allowed to attach for 24 to 48 h.

**Re-AO-BBN** uptake was assessed through live cell wide-field fluorescence microscopy. Initially, cells were washed with PBS and stained with Hoechst 33342 (0.2 µg/mL) prepared in culture medium for 30 min. Then, the culture media was replaced by FluoroBrite™ DMEM with high D-glucose (Gibco A18967-01, Grand Island, NY, USA) containing 1 µM **Re-AO-BBN** or a compound-free control. Time-lapse imaging started immediately on a Leica DMI6000 B widefield fluorescence system equipped with a HC PL APO 40 × /1.10 water immersion objective, EL6000 mercury metal halide light source, Hamamatsu Orca-Flash4.0 CMOS camera (2048 × 2048 pixels, pixel size: 6.5 × 6.5 μm) and bandpass filter cubes. 37 °C and 5% CO_2_ were maintained throughout. Time resolution was 10 min. Images were background corrected using the flat field/dark frame algorithm.

To study the intracellular location of uptaken **Re-AO-BBN**, cells were washed with PBS, incubated with MitoTracker™ Deep Red FM (1:1000, Invitrogen, Bleiswijk, The Netherlands) and **Re-AO-BBN** (1 µM) in FluoroBrite™ DMEM with high D-glucose for 30 min, fixed with 4% paraformaldehyde (in PBS) at 4 °C for 20 min, washed with PBS, incubated with Hoechst (0.2 µg/mL) for 1 h, washed and left submerged in PBS. Cells were imaged on a Leica TCS SP8 confocal microscope, equipped with a HC Plan Apo 20 × /0.75 dry objective, 405, 488 and 638 nm laser lines, PMT (MitoTracker) and HyD (Hoechst, **Re-AO-BBN**) detectors. The pinhole was set to 1 airy unit and Nyquist sampling was met in xyz (132 × 132 × 685 nm). Mitochondria accumulation was assessed by the Pearson coefficient between **Re-AO-BBN** and MitoTracker fluorescence intensities, considering an appropriate threshold.

#### 3.3.7. Clonogenic Assay

The clonogenic assay was performed for **^99m^Tc-AO-BBN** and for the rhenium conjugates **Re-TPP-BBN** and **Re-AO-BBN**, as we have previously described for **^99m^Tc-BBN**, **^99m^Tc-TPP-BBN [27]**. PC3 cells were seeded at a density of 50000 cells per well in a 24 well-plate and allowed to attach overnight. Cells were incubated with several activities (0–3.7 MBq) of **^99m^Tc-AO-BBN** and several concentrations (10^−10^–10^−7^ M) of the rhenium conjugates in 0.5 mL of culture medium, for 24 h at 37 °C. Immediately, after incubation, cells were seeded out in appropriate dilution to form colonies, in 2 weeks, with at least 50 cells. Colonies were fixed with methanol: glacial acetic acid (3:1) and stained with Giemsa (4%).

The Plating Efficiency (PE), ratio of the number of colonies to the number of cells seeded, and the Survival Fraction (SF), number of colonies that arise after treatment of cells, expressed in terms of PE, were obtained following the methodology described in [66], where:

(1) $$\mathrm{PE}=\frac{\mathrm{number}\;\mathrm{of}\;\mathrm{colonies}\;\mathrm{formed}\;}{\mathrm{number}\;\mathrm{of}\;\mathrm{cells}\;\mathrm{seeded}}\times 100\%$$

(2) $$\mathrm{SF}=\frac{\mathrm{number}\;\mathrm{of}\;\mathrm{colonies}\;\mathrm{formed}\;\mathrm{after}\;\mathrm{treatment}}{\mathrm{number}\;\mathrm{of}\;\mathrm{cells}\;\mathrm{seeded}\times \mathrm{PE}}$$

#### 3.3.8. γ-H2AX Assay and Foci Analysis

PC3 cells were seeded at a density of 10000 cells per well in an eight-well chamber slide and allowed to attach overnight. Cells were incubated with several activities (0.37, 0.74, 1.85 and 3.7 MBq) of HPLC purified **^99m^Tc-BBN**, **^99m^Tc-TPP-BBN** and **^99m^Tc-AO-BBN** in 0.3 mL of culture medium, for 24 h at 37 °C. [^99m^TcO_4_]^−^ was used as a negative control, at the same activities, since it does not efficiently internalize into cells [21] and consequently should not target the DNA to induce radiotoxic effects. PC3 cells were washed three times with PBS, and fixed with 4% formaldehyde in PBS for 15 min. After washing with PBS, cells were permeabilized with Triton X-100 (0.5%) at room temperature for 5 min followed by two washing steps with 1% BSA in PBS. Then, cells were incubated with an anti-γ-H2AX primary antibody (mouse anti-γ-H2AX (ser139), Stressgen) at 2 μg/mL for 1 h. After being washed twice with 1% BSA in PBS, cells were incubated with a Texas Red-X-conjugated anti-mouse secondary antibody at 1mg/mL for 1 h, followed by three washing steps with PBS. Cells were finally mounted in anti-fade mounting media with DAPI (Vectashield H-1200, Vector Laboratories).

Cells were analyzed under 64x magnification. Several high-quality 2D images were randomly collected in each slide and analyzed using the pipeline Speckle Count from the freeware CellProfiler [48]. At least 200 nuclei were analyzed per experiment per dose. Statistical analysis was performed with Origin 7.5 software.

#### 3.3.9. Micronuclei Assay

PC3 cells were seeded at a density of 50000 cells per well in a 24 well-plate and allowed to attach overnight. Cells were incubated with several activities (0, 0.185, 0.37, 0.74, 1.85, 3.7 MBq) of HPLC purified **^99m^Tc-BBN**, **^99m^Tc-TPP-BBN** and **^99m^Tc-AO-BBN** in 0.5 mL of culture medium, for 24 h at 37 °C. The cytokinesis-block micronucleus (CBMN) assay allows better precision because the data obtained are not confounded by altered cell division kinetics caused by cytotoxicity of agents tested or sub-optimal cell culture conditions [67]. Cytochalasin B (Sigma-Aldrich) in a final concentration of 2 µg/mL was added to the culture medium 44 h after initial incubation to inhibit cytokinesis, allowing cells to be binucleated. After 24 h of incubation with cytochalasin B (time needed to have the majority of cells binucleated), cells were harvested by centrifugation and submitted to a mild hypotonic shock (0.075 mol/L KCl solution) to enlarge the cellular cytoplasm. The cells were then smeared onto clean glass slides, allowed to dry, fixed with methanol:glacial acetic acid (3:1) and finally stained with 4% (*w/v*) Giemsa (Merck; Darmstadt, Germany).

#### 3.3.10. Assessment of mtDNA Copy Number

Cells were grown close to confluence in T25 flasks before being incubated (or not as a control) for 2 h with 9.25 MBq/mL of **^99m^Tc-BBN** and **^99m^Tc-TPP-BBN**. After incubation, the cells were detached, washed once with PBS and resuspended in 200 µL PBS. Then, total DNA was isolated using the “DNeasy Blood and Tissue Kit” (Qiagen), according to the manufacturer’s instructions. DNA concentration and purity were determined using a μDrop™ plate in a Varioskan™ LUX microplate reader (Thermo Fisher Scientific), and the samples were stored at −20 °C until further use. Only samples with high purity were used for the qPCR. qPCR was carried out in 96-well plates using the qTower3 system (Analytik Jena GmbH). Each well contained a reaction volume of 20 μL, which was composed of: 6.25 ng of total DNA, 1 × KAPA2G Fast HotStart ReadyMix (Roche), 1 × fluorescent dye EvaGreen (Biotium) and 500 nM of the forward and reverse primers (StabVida). PCR efficiencies were calculated using the qPCRsoft 4.1 (Analytik Jena GmbH) by preparing template dilutions corresponding to 12.5, 6.25, 3.125 and 1.5625 ng of total cellular DNA. The presence of the specific amplification products was confirmed through agarose gel electrophoresis. The results were used to calculate the number of copies of mitochondrial DNA as previously described [68], assuming a ploidy of 3.3 for the PC3 cell line [69].

#### 3.3.11. Determination of Cell Membrane Integrity and Cellular ATP Levels

PC3 cells were seeded at a density of 10,000 cells per well in a 96 well-plate and allowed to attach overnight. Cells were incubated with several activities (0.74 and 1.85 MBq) of HPLC purified **^99m^Tc-BBN** and **^99m^Tc-TPP-BBN** in 0.1 mL of HBSS supplemented with galactose and 1% BSA culture medium, for 2 h at 37 °C. Cells incubated only with this medium were used as a basal control and cells incubated with CCCP (10 μM) as a positive control for mitochondrial inhibition. After the 2 h, the Mitochondrial Toxglo assay was performed according to the manufacturer’s instructions (Promega). Assays were performed in triplicate.

#### 3.3.12. Statistical Analysis

All data were shown as the mean values ± standard error of the mean (S.E.M.). Statistical analysis was carried out using OriginLab software. Statistical differences between treatment and control samples were assessed using two-tailed Student’s t-test. Differences among more than two groups were assessed by one-way ANOVA followed by Tukey’s test. The threshold for statistical significance was set to *p* = 0.05.

## 4. Conclusions

To our knowledge, this work represents the first contribution reporting a comparative study of the radiobiological effects induced by structurally related targeted radioconjugates, **^99m^Tc-TPP-BBN** and **^99m^Tc-AO-BBN**, showing preferential accumulation in the mitochondria or nucleus of tumor cells. These dual-targeted radioconjugates caused a remarkably high reduction of the survival of PC3 cells when compared with the single-targeted congener **^99m^Tc-BBN**. Moreover, the dual-targeted radioconjugates led to an augmented formation of γ−H2AX foci and micronuclei. **^99m^Tc-TPP-BBN** also caused the reduction of mtDNA copy number, although enhancing the ATP production by PC3 cells. These differences can be attributed to the augmented uptake of **^99m^Tc-TPP-BBN** in the mitochondria and enhanced uptake of **^99m^Tc-AO-BBN** in the nucleus, which promoted the irradiation of these radiosensitive organelles with the short path-length AEs emitted by ^99m^Tc. In particular, the results obtained for **^99m^Tc-TPP-BBN** reinforce the relevance of targeting the mitochondria to promote stronger radiobiological effects by AE-emitting radioconjugates. Nonetheless, these effects are not necessarily localized only in the mitochondria since there is an intricate crosstalk between mitochondrial dysfunction, nuclear DNA damage and different cell death mechanisms.

In brief, our study showed that mitochondria targeting is as effective as the nuclear targeting to induce lethal radiobiological effects in tumor cells, thus deserving further attention in the design of radioconjugates for Auger therapy of cancer. These results are inspiring for us and for other researchers from this field, opening the way to apply our approach to more efficient Auger emitting radiometals (e.g., ^111^In, ^161^Tb or ^191^Pt), using other clinically relevant targeting vectors (e.g., somatostatin analogs or PSMA inhibitors) in combination with alternative cleavable linkers and/or mitotropic moieties.

## Figures and Tables

**Table 1:** Radio-HPLC retention times and log P_o/w_ values at pH 7.4 for complexes **^99m^Tc-BBN, ^99m^Tc-TPP-BBN** and **^99m^Tc-AO-BBN**.

Complex	Rt (min) ^a,b^	log P_o/w_^c^
**^99m^Tc-BBN** [9]	20.6 (20.2)	−0.75
**^99m^Tc-TPP-BBN** [9]	21.4 (20.8)	0.45
**^99m^Tc-AO-BBN**	25.0 (24.5)	−0.84

^a^ Using a gradient of aqueous 0.1 % CF_3_COOH and acetonitrile containing 0.1% CF_3_COOH. ^b^ The values in parentheses are for the congener Re complexes.^c^ At pH 7.4.

## Data Availability

Not applicable.

## Supplementary Materials

The following are available online at https://www.mdpi.com/article/10.3390/ijms23137238/s1.
