# Supplementary material for: Searching for a Paradigm Shift in Auger-Electron Cancer Therapy with Tumor-Specific Radiopeptides Targeting the Mitochondria and/or the Cell Nucleus
Source: Int J Mol Sci. 2022 Jun 29;23(13):7238. doi: 10.3390/ijms23137238 (PMC9266350; doi:10.3390/ijms23137238)
Supplement: Supplementary file 1 [file ijms-23-07238-s001.zip › ijms-1730458-supplementary.pdf]

Supplementary Material

Searching for a Paradigm Shift in Auger-Electron Cancer Therapy with Tumor-Specific Radiopeptides Targeting the Mitochondria and/or the Cell Nucleus

Célia Fernandes[1,2], Elisa Palma[1], Francisco Silva[1], Ana Belchior[1], Catarina I. G. Pinto[1], Joana F. Guerreiro[1], Hugo M. Botelho[3], Filipa Mendes[1,2], Paula Raposinho[1,2,*], António Paulo[1,2,*]

FIGURE S 1. ERROR! BOOKMARK NOT DEFINED.

FIGURE S 2. ERROR! BOOKMARK NOT DEFINED.

FIGURE S 3. 3

FIGURE S 4. 4

A)

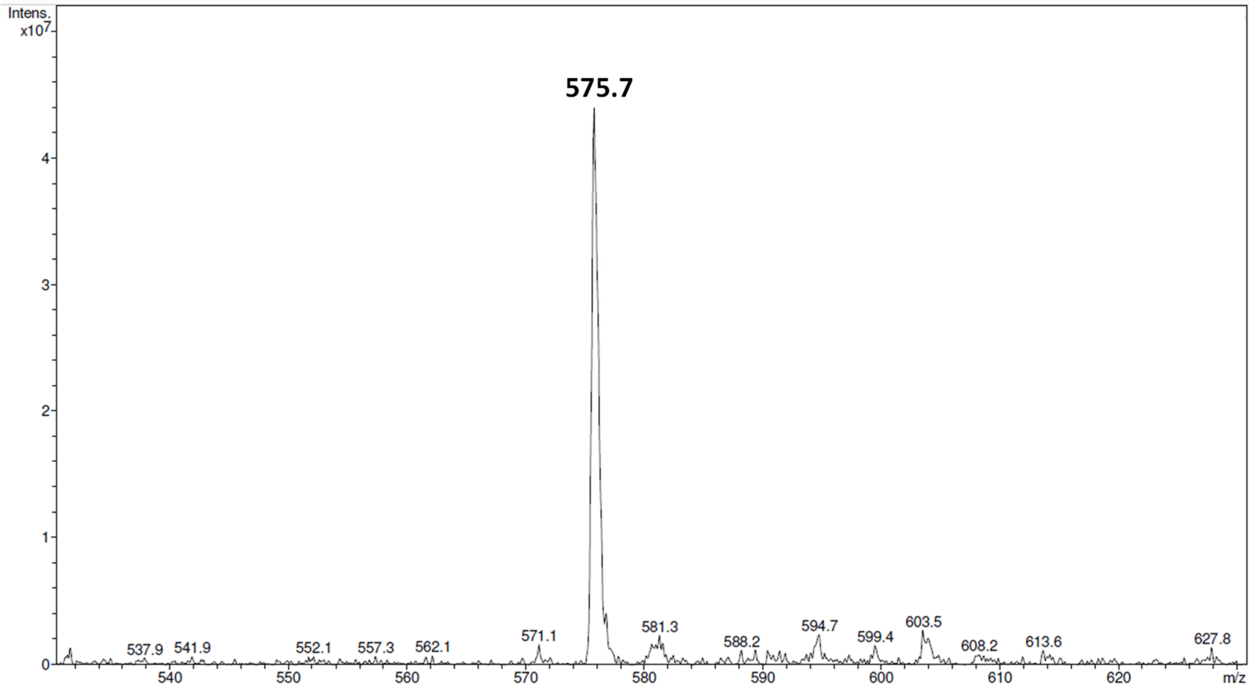

B)

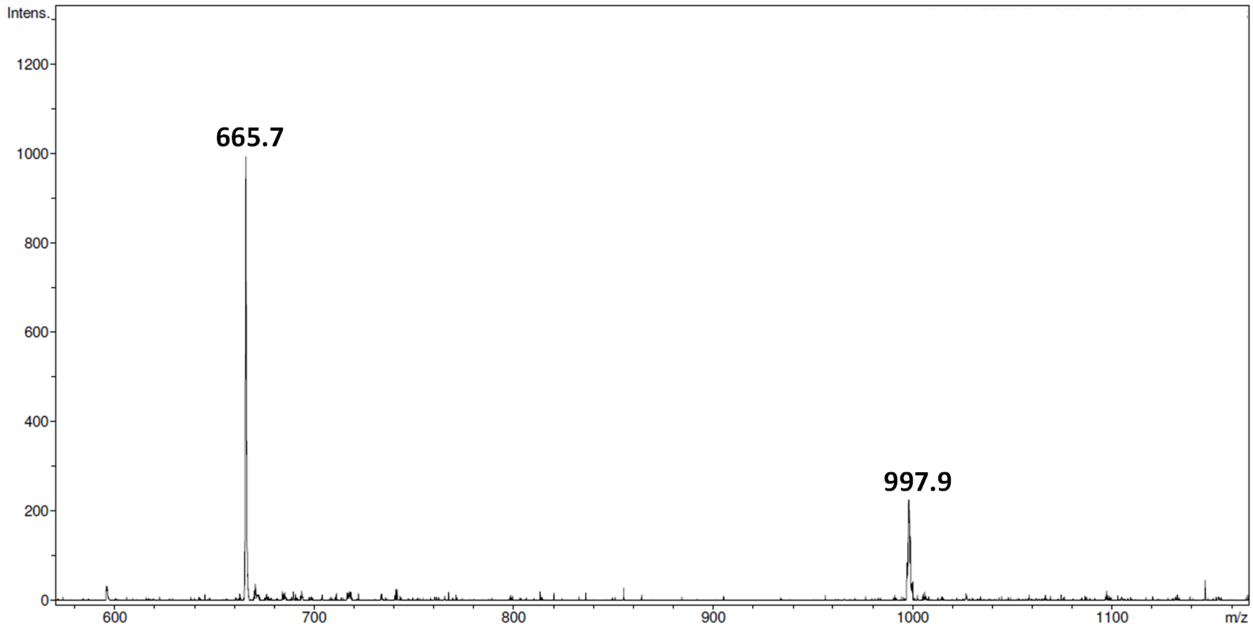

Figure S1. **A)** ESI-MS spectrum of compound **AO-Pz-BBN** (m/z calcd for $[C_{84}H_{123}N_{24}O_{14}S + 2H]^{3+}$: 575.65; found $[M + 2H]^{3+}$ 575.7); **B)** ESI-MS spectrum of compound **Re-AO-BBN** (m/z calcd for $[C_{87}H_{123}N_{24}O_{17}SRe + H]^{3+}$ = 665.29; found $[M+H]^{3+}$ 665.7), and $[C_{87}H_{123}N_{24}O_{17}SRe]^{2+}$ = 997.44; found $[M]^{2+}$ 997.9).

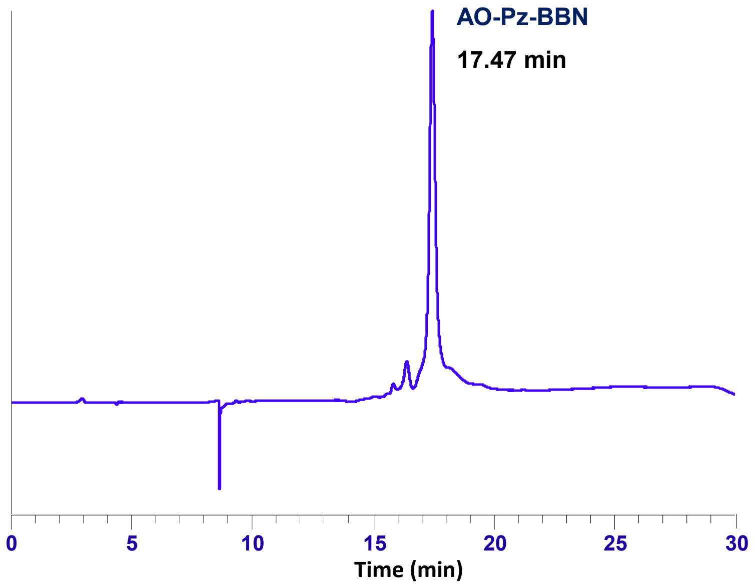

Figure S2. HPLC chromatogram (method II) of **AO-Pz-BBN** (detection at λ=254 nm).

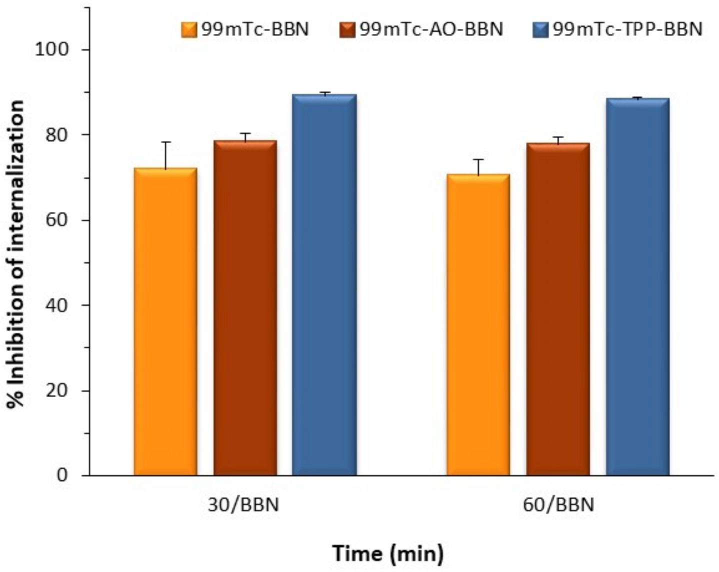

Figure S3. GRP receptor-blocking study. Inhibition of internalization of **^{99m}Tc-BBN**, **^{99m}Tc-AO-BBN** and **^{99m}Tc-TPP-BBN** by coincubation with [Tyr^4]-BBN (0.25 μg/0.5 mL/well) in PC3 cells, at 37 °C. Results were calculated from independent biological replicates (mean ± SEM, n = 4).

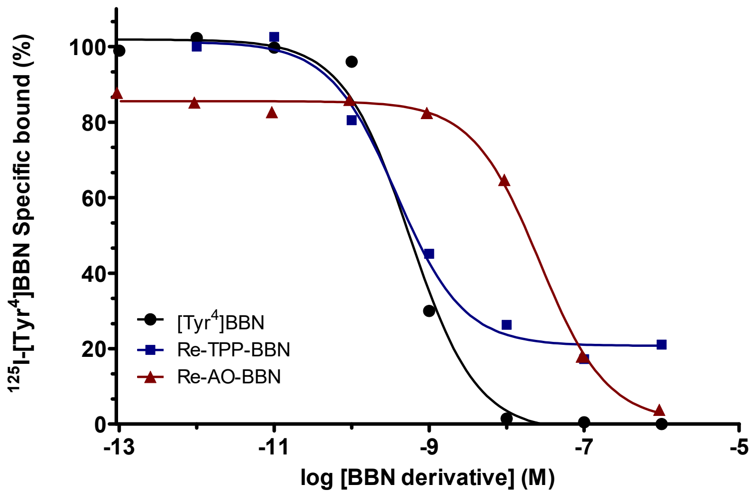

Figure S4. Competitive binding curves for BBN derivatives. *In vitro* inhibition of ^{125}I-[Tyr^4]-BBN binding to GRPR on PC3 prostate cell line by [Tyr^4]-BBN peptide (IC_{50} = 0.56 ± 0.16 nM), **Re-AO-BBN** (IC_{50} = 26.5 ± 6.8 nM) and **Re-TPP-BBN** (IC_{50} = 0.38 ± 0.14 nM). Results expressed as percent of binding were calculated from independent replicates (mean ± SEM, n = 3).
